# LncDC: a machine learning-based tool for long non-coding RNA detection from RNA-Seq data

**DOI:** 10.1038/s41598-022-22082-7

**Published:** 2022-11-09

**Authors:** Minghua Li, Chun Liang

**Affiliations:** Department of Biology, Miami University, Oxford, OH 45056 USA

**Keywords:** Computational biology and bioinformatics, Machine learning

## Abstract

Long non-coding RNAs (lncRNAs) play an essential role in diverse biological processes and disease development. Accurate classification of lncRNAs and mRNAs is important for the identification of tissue- or disease-specific lncRNAs. Here, we present our tool LncDC (Long non-coding RNA detection) that is able to accurately predict lncRNAs with an XGBoost model using features extracted from RNA sequences, secondary structures, and translated proteins. Benchmarking experiments showed that LncDC consistently outperformed six state-of-the-art tools in distinguishing lncRNAs from mRNAs. Notably, the use of sequence and secondary structure (SASS) k-mer score features and flexible ORF features improved the classification capability of LncDC. We anticipate that LncDC will definitely promote the discovery of more and novel disease-specific lncRNAs. LncDC is implemented in Python and freely available at https://github.com/lim74/LncDC.

## Introduction

Advancement in next-generation sequencing (NGS) technology, especially transcriptome profiling, enabled the discovery of thousands of novel RNA transcripts. Among them, a class of RNA transcripts named long non-coding RNAs (lncRNAs) obtained special attention. lncRNAs are RNA transcripts longer than 200 nucleotides (nt) without protein-coding capabilities^1^. Similar to messenger RNAs (mRNAs), lncRNAs are typically transcribed by RNA polymerase II so that most of them have 5’-end caps, 3’-end poly(A) tails, alternative splicing, and are subjected to other post-transcriptional processes^2^. However, compared with mRNAs, lncRNAs are usually less evolutionarily conserved and have lower expression levels, making them difficult to be identified accurately^3^. In addition, lncRNAs are expressed more tissue- or condition-specific than mRNAs indicating that there are still abundant lncRNAs to be discovered in different tissues and with different conditions^4^.

Even though lncRNAs do not code for proteins, they are involved in diverse biological processes that regulate the expression of protein-coding genes, such as chromosome modification, transcription machinery interfering, gene-dosage compensation, and enhancer-associated activation^5^. Also, lncRNAs can bind proteins to form lncRNA-protein complexes, leading to signaling pathways alternation or mRNA splicing modification^5^. The complicated functions of lncRNAs and their broad involvement in different biological processes make them a research hotspot in biomedicine.

lncRNAs are involved in diverse disease conditions such as neurodegenerative diseases, cardiovascular diseases, and cancer^6–9^. In particular, lncRNAs become new diagnostic biomarkers and potential therapeutic targets for cancer treatment because of their aberrant expression in different cancer cells. For instance, the prostate cancer-specific lncRNA gene prostate cancer antigen 3 (PCA3) is overexpressed in prostate cancer cells but can barely be found in other cancers. As an effective diagnostic biomarker for prostate cancer, highly expressed PCA3 can be detected from urine samples of prostate cancer patients^10^. Although researchers have already identified many previously unannotated lncRNAs, some of which are cancer-specific, more disease-specific lncRNAs are yet to be identified.

RNA-Seq is commonly used to identify novel lncRNA transcripts because of its high throughput characteristic and sensitivity in detecting lowly expressed RNA transcripts. Wet-lab approaches reverse transcription polymerase chain reaction (RT-PCR) and mass spectrometry (MS) of peptides are always required for the validation of newly discovered lncRNAs^11,12^. Although evidence from RT-PCR and MS is reliable, such validation is labor-intensive and time-consuming when many lncRNA candidates need to be identified. Besides wet-lab approaches, alignment-based homology search in protein databases of multiple species is also used to identify lncRNAs, such as coding-potential calculator (CPC)^13^. However, alignment-based methods are adept in evaluating protein coding potential for interspecific or intraspecific conserved RNA transcripts such as mRNAs but not suitable for lncRNAs because they are mainly species-, tissue-, and/or condition-specific. For example, the GENCODE project v7 catalog of human lncRNA shows that near 30% of the identified lncRNA transcripts are primate-specific^11^. In addition, lncRNAs that are partially overlapped with protein-coding genes can easily be misclassified as mRNAs through alignment-based homology search methods^14,15^.

Several alignment-free methods or tools have been developed to overcome the flaws of the alignment-based approaches. Alignment-free methods mainly extract sequence-derived features from mRNAs and lncRNAs and apply different machine learning algorithms to distinguish them. CPAT differentiates mRNAs and lncRNAs by using a logistic regression model with several features, including open reading frame (ORF) length, ORF coverage, Fickett score, and hexamer usage bias^14^. If an RNA has a long ORF, it usually means that it has a high probability of being translated into a protein, even though studies suggest that some small ORFs can encode micro peptides of less than 100 amino acids in length^16^. Fickett score is a linguistic feature used to measure the variety of nucleotide positions and compositions between mRNAs and lncRNAs^17^. Hexamer usage bias is a measurement that distinguishes mRNAs and lncRNAs based on the fact that a hexamer in the longest ORF of a mRNA can decide the adjacent amino acids in the translated peptide^14,18^. PLEK applies a support vector machine (SVM) model with an improved k-mer scheme to predict lncRNAs and mRNAs, where the k-mer patterns indicate a specific string order with k nucleotides in an RNA transcript. CPC2 is an upgraded version of CPC, which integrated a SVM model with Fickett score, ORF length, ORF integrity, and protein pH isoelectric point (PI) features to identify lncRNAs^19^. Besides the features mentioned ahead, CPPred proposed a feature combination named CTD features, which describe the nucleotide composition and distribution in an RNA transcript^20^. LncFinder is an R package that applies ORF, secondary structure, and physicochemical property-based features to identify lncRNAs^21^. COME integrates sequence-derived and experiment-based features for lncRNA identification, such as GC content, sequence conservation, expression abundance and histone modification state^22^.

Although current machine learning-based tools have encouraging performances in lncRNA identification, they still have some drawbacks. First, most of them heavily rely on the longest ORF of an RNA transcript. However, in the gold-standard NCBI RefSeq manually curated gene annotations, there are 1909 mRNA transcripts whose coding sequence (CDS) differ from the longest ORF, indicating that features extracted from the longest ORF do not always accurately represent the properties of CDS^23^. Due to the difference between the longest ORF and CDS, these mRNAs are easily misclassified as lncRNAs. Second, RNA transcripts are capable of forming specific secondary structures. Those secondary structures contribute to the functions of RNA transcripts, such as transcription and translation factors targeting, scaffolding for RNA–protein complexes in nuclei, or interfering with post-translational modification of proteins in cytoplasm^24,25^. The secondary structures that mRNAs and lncRNAs formed can be different, but few tools use secondary structure-based information to aid classification of the two types of RNA. Third, most of the current tools apply a random under-sampling strategy for balancing training data, which randomly eliminates several majority class examples. For instance, lncFinder randomly selects 8,000 lncRNAs and 8,000 mRNAs from the GENCODE database to construct the training dataset of human, although the number of mRNAs in this database far exceeds the number of lncRNAs^11,21^. Random under-sampling is easy to perform, but some data that may have an important impact on classification may be lost, thereby reducing the performance of model prediction^26^.

Here, we developed an alignment-free, machine learning-based tool named LncDC, which integrates sequence intrinsic features (SIFs), secondary structure features (SSFs), and protein features (PFs) with the XGBoost algorithm to detect lncRNAs from RNA-Seq data. In order to fully capture the characteristics of CDS, we defined four types of ORF similar to those described in FEELnc^27^. We also designed novel secondary structure features that integrate both primary sequence and secondary structure information from RNA transcripts. For each RNA transcript, we translated it to a predicted protein and extracted PFs from the protein sequence. We used recursive feature elimination with cross-validation (RFECV) for feature selection, which prunes a single feature in each step and stops until the performance of model prediction does not drop any more^28^. During model training, we used synthetic minority over-sampling technique (SMOTE) to reduce loss of potentially valuable data by introducing new minority class samples between adjacent minority class examples^29^. We benchmarked the performance of LncDC against six existing tools (CPAT, PLEK, CPC2, CPPred, LncFinder, and COME), and the results showed that LncDC outperformed these state-of-the-art tools on both human and mouse testing datasets. LncDC allows researchers to train customized models with their own data, with or without secondary structure features. LncDC can also automatically balance training data so that researchers can use datasets with large differences in the number of mRNAs and lncRNAs to train models. LncDC is mainly used for the identification of lncRNAs in humans, mice, and other closely related species.

Osteosarcoma (OS) is the most common primary malignancy that starts in bones. It usually occurs in children and adolescents, while individuals older than 60 also have a high occurrence rate^30^. Metastasis is the primary cause of OS-associated death, and the 5 year survival rate of OS patients diagnosed with metastasis is less than 20%^31^. At present, the diagnosis of OS relies on X-ray, computed tomography (CT) scan, magnetic resonance imaging (MRI), and biopsy, and the treatment of OS includes radiation, surgery, and chemotherapy^32^. Human epidermal growth factor receptor 2 (HER2) as a biomarker was proposed to be a treatment target because of its abnormal expression in OS cancer cells and association with lung metastasis, but the drug targeted to HER2 didn’t improve the survival rate of OS patients significantly^33,34^. In contrast to protein biomarkers, recent studies show that lncRNAs become promising biomarkers or therapeutic targets for OS, such as fibroblast growth factor receptor 3 antisense transcript 1 (FGFR3-AS1), hypoxia-inducible factor-2α promoter upstream transcript (HIF2PUT), and taurine upregulated gene 1 (TUG1)^35^. For instance, FGFR3-AS1 is upregulated in OS cancer cells and correlated with metastasis and poor prognosis. Knockdown of FGR3-AS1 inhibits both the proliferation of OS cells in vitro and the growth of xenograft tumors in vivo^36^. Overexpression of HIF2PUT decreases the growth rate, migration, and sphere-forming ability of OS cancer cells possibly due to its negative regulation to transcription factor hypoxia-inducible factor-2α (HIF2α)^37^. To discover more novel lncRNA biomarkers or therapeutic targets, we developed a bioinformatics pipeline that combines LncDC and other popular bioinformatics tools to detect novel OS-specific lncRNAs from 180 OS RNA-Seq data obtained from the therapeutically applicable research to generate effective treatments (TARGET) database (https://ocg.cancer.gov/programs/target). In total, we identified 97 novel OS-specific lncRNA transcripts in OS tissues by this pipeline, which were previously unannotated and were not detected in adjacent normal bone tissues. These newly identified lncRNAs with high confidence expand the annotation of OS transcriptome and provide potential biomarkers or therapeutic targets for OS treatment.

## Results

### Data distribution

**Figure 1:**
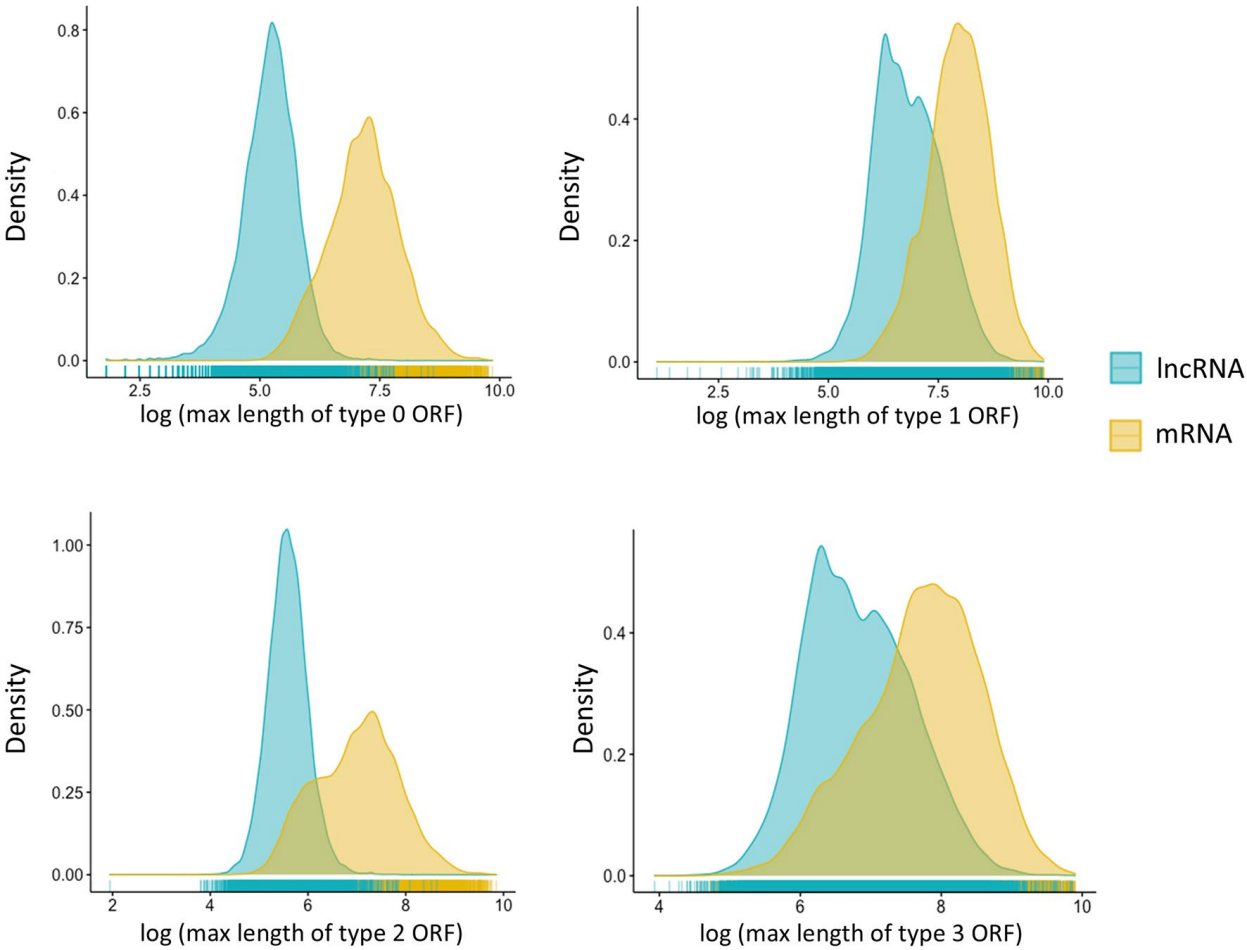
The distribution of the max length of four types (type 0, 1, 2, and 3) of ORFs for lncRNAs and mRNAs in H-Train. The x-axis is the log of max length of ORFs, and the y-axis is density.

To overcome the drawback that the longest, conventionally defined ORF of many mRNAs is different from their annotated CDS, we defined four types of ORFs (Supplementary Fig S1). We extracted all the ORF-associated features from each ORF category, such as max ORF length, relative codon bias, and hexamer score. As shown in Fig. 1, the logarithm transformed values of max length of type 0 ORF, which is the conventionally defined ORF, were higher in mRNAs and lower in lncRNAs within the human train dataset (H-Train). As extra features in addition to type 0 ORF, the values of the max lengths of type 1, type 2, and type 3 ORFs also showed distinctive distributions between mRNAs and lncRNAs although their patterns were slightly different from that of the type 0 ORF. The hexamer scores and relative codon bias of distinct ORF types also formed into two clusters for mRNAs and lncRNAs, respectively (see Supplementary Fig. S2 and S3).

**Figure 2:**
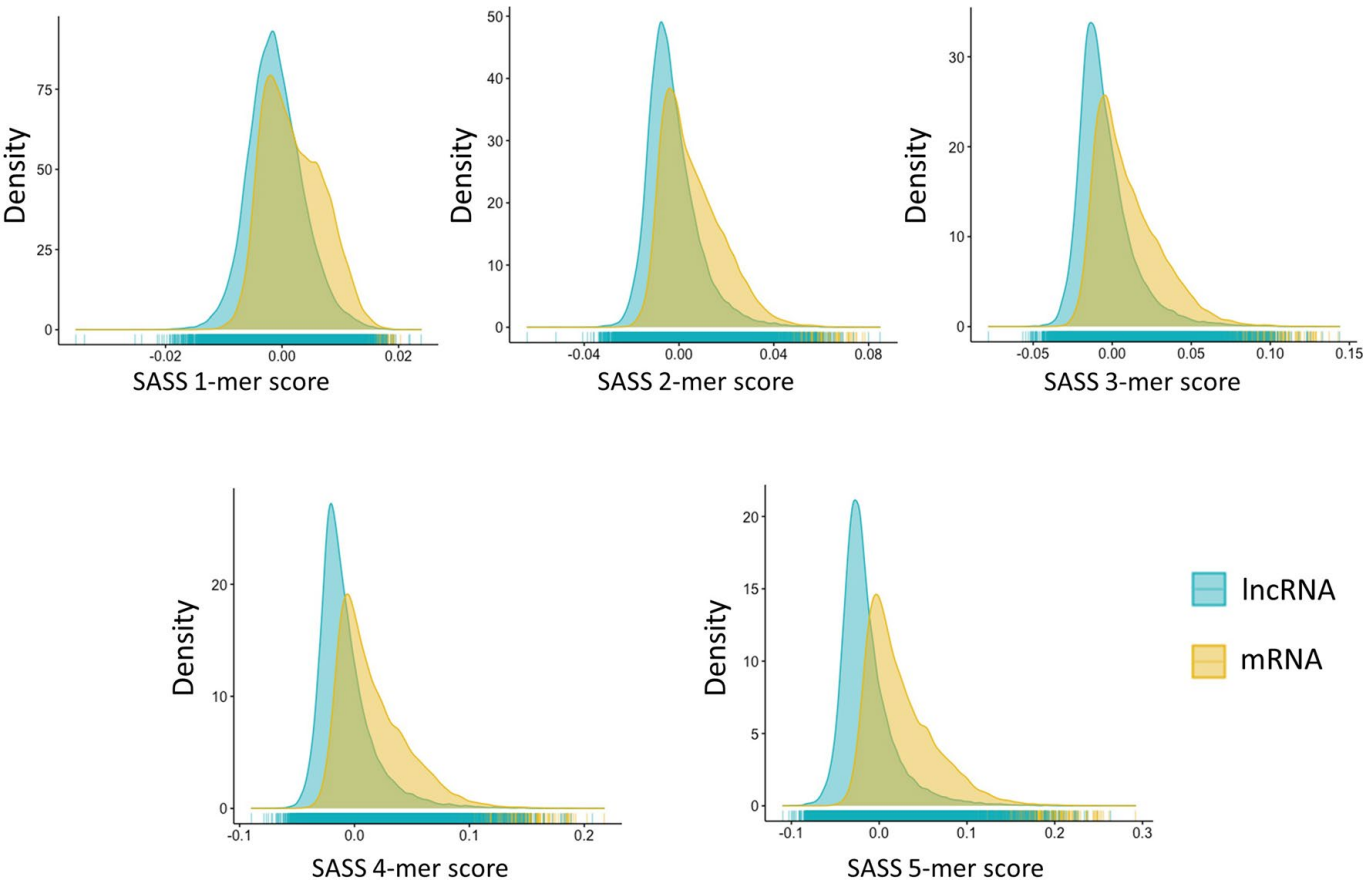
The distribution of the sequence and secondary structure (SASS) k-mer scores for lncRNAs and mRNAs in H-Train. The x-axis is the k-mer score, and the y-axis is density.

Moreover, we designed sequence and secondary structure (SASS) k-mer scores to capture the relationship between an RNA primary sequence and its secondary structure. SASS k-mer scores were concordantly higher in mRNAs but lower in lncRNAs within H-Train (Fig. 2). The pattern became more pronounced when k was larger. The explicit two clusters indicated that SASS k-mer scores have the ability to display differences in the primary sequence and secondary structure of mRNAs and lncRNAs.

### Model selection

We first compared performances of different models with a total of 57 extracted features. We used H-Train to train models with distinctive algorithms, including logistic regression (LR), decision tree (DT), support vector machine (SVM), random forest (RF), and XGBoost. For each model, a tenfold cross-validation was applied for parameter tuning. The performances of different models on the human test dataset (H-Test) are displayed in Table 1.

**Table 1:** Performance of different models on H-Test.

	$\text{Sensitivity}$	$\text{Specificity}$	$\text{Precision}$	$\text{Accuracy}$	$\text{F - score}$	$\text{MCC}$
LR	0.9745	0.9693	0.9694	0.9719	0.9719	0.9438
DT	0.9714	0.9623	0.9626	0.9668	0.9670	0.9337
SVM	0.9859	0.9647	0.9654	0.9753	0.9755	0.9508
RF	0.9813	0.9685	0.9688	0.9749	0.9750	0.9498
XGBoost	**0.9861**	**0.9740**	**0.9743**	**0.9800**	**0.9801**	**0.9601**

Bold values correspond to the highest values of each metric.

As shown in Table 1, the XGBoost model achieved the best performance than others in all criteria. The XGBoost model had the highest accuracy, 0.9800. The accuracy scores of SVM and RF were lower than XGBoost: 0.9753 and 0.9749. The DT model had the lowest accuracy, which is 0.9668. The F-score and matthews correlation coefficient (MCC) values of the SVM and RF models were very close, but both were lower than the XGBoost model. Supplementary Fig. S4 presented the receiver operating characteristic (ROC) curves of different models on H-Test. The area under the curve (AUC) score of the XGBoost model achieved the highest, 0.9974, among all the models. Because of the excellent performance of the XGBoost model, we used it in our LncDC program.

### Feature selection

**Figure 3:**
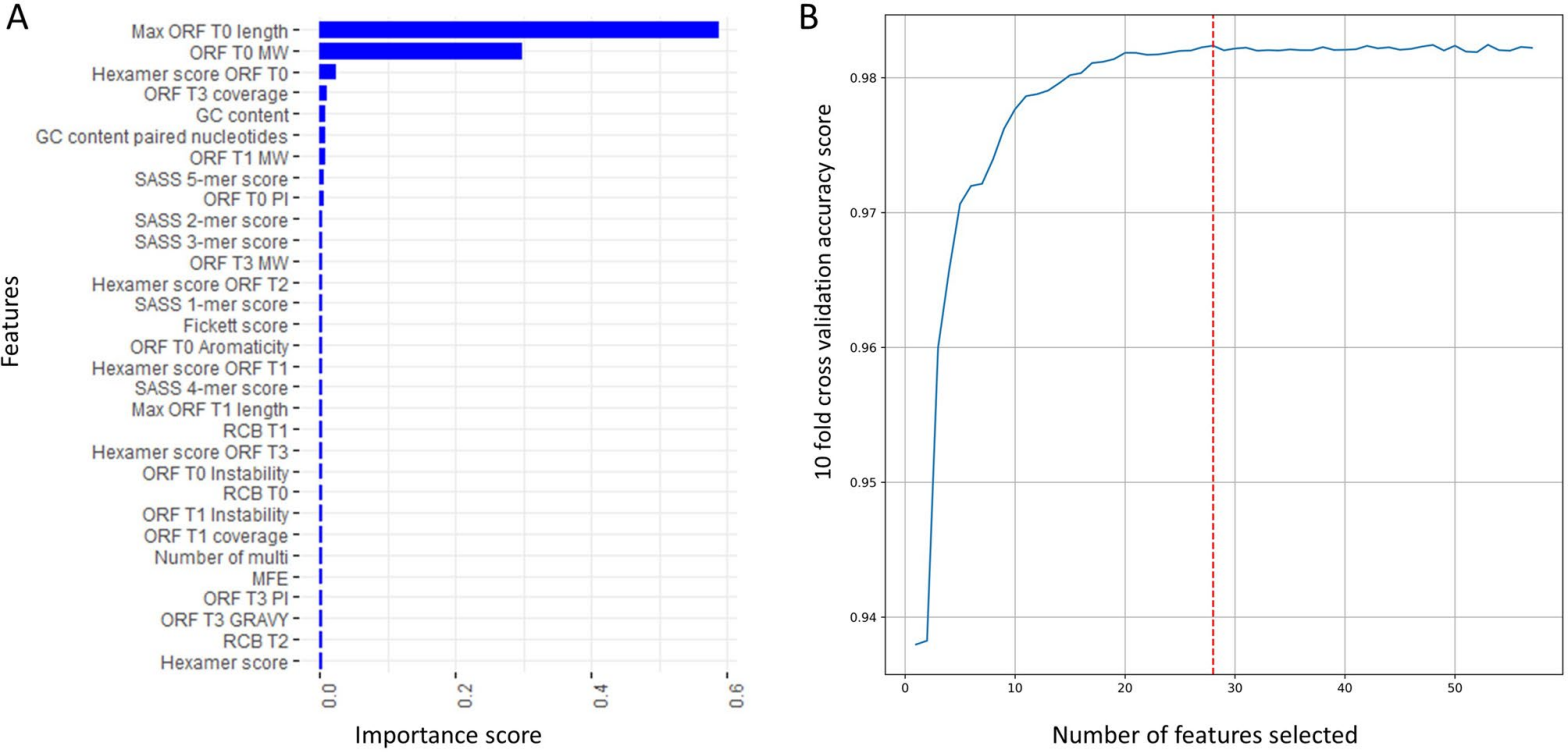
(**A**) Importance scores of the features obtained from the XGBoost model trained with H-Train. Features are listed in descending order of their importance scores, and only the top 30 features are shown in the figure. (**B**) Feature selection by RFECV on H-Train. The x-axis indicates the number of selected features, and the y-axis shows the tenfold cross-validation accuracy score for each feature subset. The red dash line indicates the peak of the accuracy score and the corresponding 28 features selected by RFECV.

At first, we examined the feature importance by gain, which represents the relative contribution of a feature for predicting binary categories by the XGBoost model. As shown in Fig. 3A, ‘Max ORF T0 length’ ranked the highest among all features, as expected. ‘ORF T0 MW’ ranked the second highest, indicating that the molecular weight of the proteins translated from type 0 ORFs in mRNAs and lncRNAs are significantly different. ‘Hexamer score ORF T0’ that measures six adjacent nucleotides components in type 0 ORFs of mRNAs and lncRNAs ranked the third. ‘ORF T3 coverage’, as a complementary feature in addition to type 0 ORF features, ranked fourth. Surprisingly, the ‘GC content’ feature ranked fifth, suggesting that the nucleotide preference of mRNAs and lncRNAs are distinct. In the case of SSFs, we could see that ‘GC content of paired nucleotides’ and ‘SASS 5-mer score’ ranked sixth and eighth among all features while other SASS k-mer score features were among the top twenty features. Such results indicated that the selected ORF features and SSFs are good predictors for XGBoost model trained with H-Train.

Next, we evaluated tenfold cross-validation accuracy scores of different feature subsets on H-Train to select the best feature subset. The cross-validation accuracy scores of various feature subsets are shown in Fig. 3B. Although the feature subset with the top 53 features had the highest tenfold cross-validation accuracy score, 0.9824, the feature subset with the top 28 features had an accuracy score, 0.9823, only 0.0001 lower. To select the most important features and remove redundant ones, we used the feature subset with the top 28 features in the LncDC program for model training and prediction. We also tested both the top 53 feature subset and the top 28 feature subset on H-Test and evaluated their performances. Interestingly, the accuracy score of the top 28 feature subset, 0.9799, was very close to the accuracy of the top 53 feature subset, 0.9802; but the specificity and precision were higher in the top 28 feature subset (Supplementary Table S1). This also suggested that some of the total extracted features were redundant, and the top 28 feature subset adequately captured the major differences between mRNAs and lncRNAs.

Among the 28 features selected (Supplementary Table S2), half of them came from SIFs, which were ‘GC content’, ‘ Fickett score’, ‘Max ORF T0 length’, ‘Max ORF T1 length’, ‘Max ORF T2 length’, ‘ORF T0 coverage’, ‘ORF T1 coverage’, ‘ORF T3 coverage’, ‘Hexamer score ORF T0’, ‘Hexamer score ORF T1’, ‘Hexamer score ORF T2’, ‘Hexamer score ORF T3’, ‘RCB T0’ and ‘RCB T1’. There were 6 SSFs in the selected feature subset: ‘SASS 1-mer score’, ‘SASS 2-mer score’, ‘SASS 3-mer score’, ‘SASS 4-mer score’, ‘SASS 5-mer score’, and ‘GC content of paired nucleotides’. The remaining 8 features were PFs, including ‘ORF T0 PI’, ‘ORF T0 MW’, ‘ORF T0 aromaticity’, ‘ORF T0 instability’, ‘ORF T1 MW’, ‘ORF T1 instability’, ‘ORF T2 MW’ and ‘ORF T3 MW’.

Moreover, we examined if the 6 selected SSFs contribute to the overall performance of the model. After excluding the 6 selected SSFs, the performance of the remaining 22 chosen features (top 22 features) was evaluated on H-Test. It showed that the overall performance of the model was dropped once the 6 selected SSFs were removed (Supplementary Table S1). The accuracy score decreased from 0.9799 to 0.9783, precision dropped from 0.9786 to 0.9746, MCC went down from 0.9598 to 0.9567 and the specificity declined from 0.9786 to 0.9745. In contrast, the sensitivity slightly raised from 0.9812 to 0.9822, indicating that the top 22 feature subset without SSFs can detect more lncRNAs but with more false positives.

At last, we investigated if the 6 selected SSFs themselves also have a solid ability to classify lncRNAs and mRNAs. As shown in Supplementary Table S1, the accuracy score of the 6 SSFs subset on the H-Test dataset was 0.8680, which was lower than that of the top 28 feature subset and the top 22 feature subset without SSFs but still acceptable. Surprisingly, the precision of the 6 selected SSFs subset was close to 0.90, suggesting that within the lncRNAs predicted by these features, a large proportion of them were true lncRNAs.

### Benchmarking LncDC with other popular tools

#### Performance evaluation on H-Test

**Figure 4:**
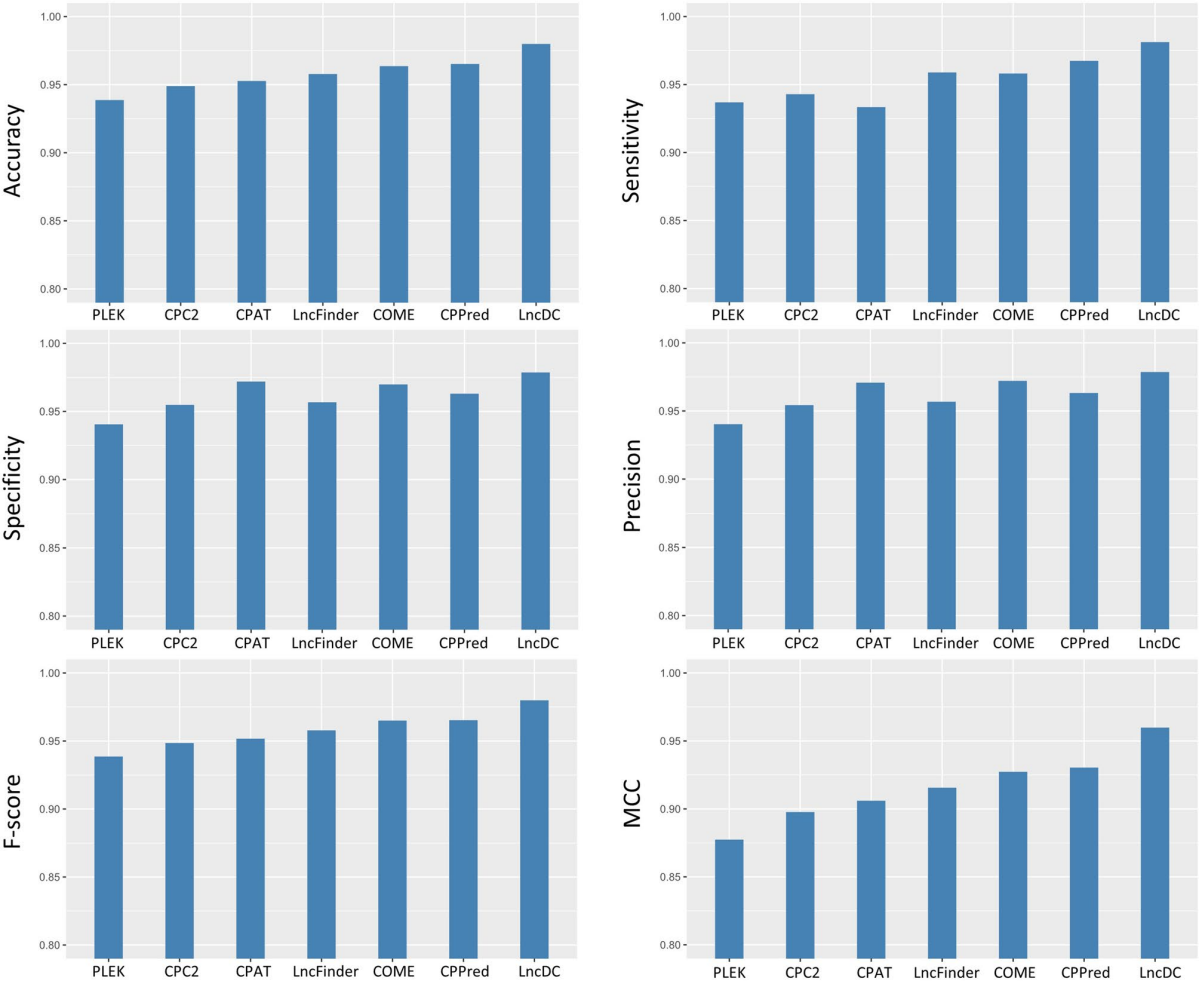
The x-axis shows the names of the tools and the y-axis shows the values of the tools in various performance metrics, including accuracy, sensitivity, specificity, precision, F-score, and MCC.

To investigate whether LncDC outperforms other popular tools, we evaluated the performances of different tools on H-Test (Fig. 4). The accuracy of PLEK was the lowest among all tools tested, which was 0.9387. The F-score and MCC value of PLEK were also lower than other tools. Compared with PLEK, CPC2 had a higher accuracy and MCC score. CPAT surpassed CPC2 with an accuracy of 0.9527. CPAT also achieved relatively high specificity and precision, 0.9719 and 0.9708, but sensitivity was relatively low, 0.9334, indicating that a large proportion of lncRNAs was misclassified as mRNAs by CPAT. Since CPAT allows users to train models by themselves, we trained a CPAT model with H-Train data and then evaluated the performance of the re-trained model in H-Test. The result showed that the accuracy of the re-trained CPAT model was slightly improved from 0.9527 to 0.9578. The specificity and precision were lower than the default CPAT model. Still, the sensitivity was higher, suggesting that the re-trained CPAT model had a better balance between sensitivity and precision. LncFinder had the same accuracy as the re-trained CPAT model. lncFinder had a good balance between sensitivity and precision, but none of those scores were higher than 0.96. COME surpassed LncFinder with an accuracy of 0.9636 and a MCC score of 0.9273. The specificity, precision, and F-score of COME were also higher than PLEK, CPC2, and LncFinder. The performance of CPPred was similar to COME in accuracy and F-score. The accuracy of CPPred and COME were 0.9652 and 0.9636, respectively. CPPred’s F-score was 0.9653 and COME’s F-score was 0.9650. The main difference between COME and CPPred was that COME had higher specificity and precision, while CPPred had higher sensitivity.

LncDC achieved the best performance than all other tools tested (accuracy, 0.9799; sensitivity, 0.9812; specificity, 0.9786; precision, 0.9786; F-score, 0.9799; and MCC, 0.9598). The very high accuracy score indicated that LncDC had a powerful discriminative capability in classifying lncRNAs and mRNAs in H-Test. We also investigated the performance of LncDC when SSFs were excluded, and the result showed that the sensitivity score was slightly increased to 0.9822, but the accuracy, precision, and MCC scores were dropped to 0.9783, 0.9746, and 0.9567, respectively, indicating that SSFs were important to the overall performance of LncDC especially in precision. Surprisingly, LncDC had the highest MCC score than other tools, which is about 8% higher than PLEK and 4% higher than LncFinder.

#### Performance evaluation on M-Test

Moreover, we evaluated the performances of different tools on the mouse test dataset (M-Test) (Table 2). The accuracy of PLEK was 0.8965, which was the lowest among all tools. CPC2 outperformed PLEK with an accuracy of 0.9476 and a MCC score of 0.8952. COME had a similar performance with CPC2 in accuracy and MCC, but COME had a higher sensitivity. The accuracy of CPAT, 0.9615, was higher than CPC2 and COME, and the MCC score was also improved to be 0.9230. Similarly, we re-trained a CPAT model with the mouse train dataset (M-Train) and then evaluated the performance of the re-trained model on M-Test. The result showed that the re-trained CPAT model had a dropped accuracy from 0.9615 to 0.9538. The specificity and precision of the re-trained CPAT model increased from 0.9628 and 0.9627 to 0.9730 and 0.9719, respectively. In contrast, the sensitivity of the re-trained CPAT model was much lower than that of the default CPAT model. LncFinder, whose accuracy score was 0.9620 and MCC score was 0.9240, was higher than CPAT. The sensitivity, specificity, and precision of LncFinder were well balanced. CPPred had an accuracy of 0.9613 and a MCC score of 0.9227, which were lower than those of CPAT and LncFinder, but its sensitivity was slightly higher.

**Table 2:** Performance of different tools on M-Test.

	$\text{Sensitivity}$	$\text{Specificity}$	$\text{Precision}$	$\text{Accuracy}$	$\text{F - score}$	$\text{MCC}$
PLEK	0.9080	0.8850	0.8876	0.8965	0.8977	0.7932
CPC2	0.9440	0.9512	0.9508	0.9476	0.9474	0.8952
COME	0.9550	0.9364	0.9379	0.9457	0.9463	0.8916
CPAT	0.9602	0.9628	0.9627	0.9615	0.9614	0.9230
CPAT (re-trained)	0.9346	0.9730	0.9719	0.9538	0.9529	0.9083
LncFinder	0.9600	0.9640	0.9639	0.9620	0.9619	0.9240
CPPred	0.9690	0.9536	0.9543	0.9613	0.9616	0.9227
LncDC (mouse model)	0.9704	**0.9812**	**0.9809**	**0.9758**	**0.9756**	**0.9516**
LncDC (mouse model, no SSFs)	0.9726	0.9752	0.9751	0.9739	0.9738	0.9478
LncDC (human model)	0.9700	0.9746	0.9744	0.9723	0.9722	0.9446
LncDC (human model, no SSFs)	**0.9742**	0.9718	0.9718	0.9730	0.9730	0.9460

Bold values correspond to the highest values of each metric.

For LncDC, we trained a model with M-Train, named it as LncDC mouse model, and then evaluated its performance on M-Test. The result showed that the LncDC mouse model achieved the best performance with an accuracy of 0.9758 and a MCC score of 0.9516. We also investigated the performance of the LncDC mouse model when SSFs were removed. It showed that the overall performance of the LncDC mouse model without SSFs was slightly worse than the LncDC mouse model; especially, the precision was decreased from 0.9809 to 0.9751.

Next, we were curious about if the LncDC human model is robust or not in mammalian lncRNA prediction, such as mice. We evaluated the performance of the LncDC human model on M-Test, and the result showed that the human model also had an excellent performance. Although compared to the LncDC mouse model, the accuracy and MCC scores of the LncDC human model were dropped from 0.9758 and 0.9516 to 0.9723 and 0.9446, respectively, it still outperformed other tools. The performance of the LncDC human model without SSFs was also evaluated. Compared to the LncDC human model with SSFs, the human model without SSFs had slightly higher sensitivity but lower specificity and precision.

#### Identification of OS-specific novel lncRNAs from RNA-seq data

After genome mapping and assembling, a total of 704,690 and 721,219 assembled RNA transcripts were obtained from Route 1 and Route 2 approaches of our bioinformatics pipeline, respectively (Supplementary Fig S5). Then we filtered out the RNA transcript assemblies that didn’t meet the criteria we set, such as shorter than 200 nt, exon number less than two, and location codes other than ‘U’, ‘I’, ‘O’ or ‘X’. In addition, we selected RNA transcript assemblies with the same genomic positions predicted by both Route 1 and Route 2 approaches to build the common set, followed by removing currently annotated RNA transcripts in various databases and the assembled RNA transcripts from normal tissues of OS patients. Finally, we obtained 101 novel RNA transcript candidates that were OS-specific.

LncDC predicted that 97 out of 101 were lncRNAs and 4 were mRNAs. The 97 OS-specific novel lncRNA transcripts came from 94 lncRNA genes where one gene ‘XLOC_195408’ in chromosome 20 had four transcript isoforms (Supplementary Fig S6A, S7A and S7B). The average length of the 97 lncRNA transcripts was around 1362 nt and the longest one was 8360 nt in size. As shown in Supplementary Fig S6B, more than half of the novel lncRNA transcripts, 58 in total, had two exons, while fewer of them had three or more exons (three exons: 25, four exons: 11, and five exons: 3). In addition, Supplementary Fig S6C showed that most of the novel lncRNA transcripts were located in the intergenic region (with a code ‘U’). In contrast, a small proportion of them overlapped with reference annotated exons on the same strand (with a code ‘O’) or on the opposite strand (with a code ‘X’).

The newly identified lncRNA transcripts with ‘X’ or ‘O’ codes overlapped with known genes, such as protein-coding genes and pseudogenes. For instance, the lncRNA transcript located on chr11:106,620,650—106,782,318 overlaps with a protein-coding gene GUCY1A2 on the opposite strand, implying that it might be an antisense lncRNA. The GUCY1A2 gene was reported to be upregulated in gastric cancer tissues and is associated with a poor prognosis^38^. The expression of the above novel antisense lncRNA in OS tissues may regulate the expression of GUCY1A2 and contribute to the progression of OS. On the other hand, pseudogenes are always believed to be inactive genes due to mutations. However, a proportion of pseudogenes has been reported to be transcribed in cells and regulate gene expression^39^. Our newly identified lncRNA transcript on chr3:80,051,883 – 80,312,353 overlapped with the pseudogene OSBPL9P1 on the same strand, suggesting a potential new RNA isoform from this gene. At last, the newly identified lncRNA transcripts with ‘U’ code were also referred to as long intergenic non-coding RNAs (lincRNA) in some literatures because of the intergenic location.

## Discussion

With widespread applications of sequencing techniques in cancer research, a large amount of RNA-Seq data has been generated and accessible to researchers. In addition to the measurement of gene expression, those RNA-Seq data are also treasures for discovering novel RNAs, especially cancer-specific RNAs. lncRNA, as a large category of RNAs, plays a considerable role in various biological processes, such as gene regulation and chromosome modification. Due to somatic mutation in cancer genomes, some lncRNAs are possibly dysregulated and highly expressed in a cancer-specific manner. Those newly discovered cancer-specific lncRNAs will potentially contribute to cancer diagnosis and therapy.

The conventionally defined ORF was used in most lncRNA identification tools, such as CPC2, CPAT, LncFinder, and CPPred, because the longest ORF of a mRNA resembles its CDS closely. However, current manually curated mRNA annotations showed that the longest ORF is different from the annotated CDS in many mRNAs. The three additional ORF categories utilized in LncDC were able to capture the CDS characteristics of mRNAs comprehensively. Although FEELnc first proposed similar ORF definitions, it only extracted a few features including ORF length, ORF coverage, and multi k-mer frequencies of different ORF types, which demonstrated limited power in discriminating mRNAs and lncRNAs^27^. On the contrary in LncDC we extracted max ORF length, hexamer score, and relative codon bias features from the additional defined ORFs, and those features displayed two distinguished clusters for mRNAs and lncRNAs in H-Train although they were in different patterns comparing to the conventionally defined ORF (Fig. 1, Supplementary Fig S2 and S3). The ORF-associated features were among the top-ranked features that contribute to the XGBoost model prediction in H-Train (Fig. 3A). They were among the 28 features selected by RFECV as well (Supplementary Table S2). The results showed that different types of ORF and their associated features effectively improved the ability of LncDC in capturing CDS characteristics of mRNAs and discriminated them from lncRNAs.

Among SSFs, our newly designed SASS k-mer scores integrated primary sequence and secondary structure information from RNA transcripts. As the number of k increased, the distinct distribution of mRNA and lncRNA SASS k-mer scores became more apparent (Fig. 2). Moreover, all of the SASS k-mer scores were selected by RFECV and were among the top 28 features. According to the experiments, the accuracy of LncDC had improved from 0.9783 to 0.9799 with the 6 selected SSFs added (Supplementary Table S1). Although the enhancement seems not a giant step, the LncDC model with only the 6 selected SSFs performed well in H-Test (precision:0.8929, specificity:0.8998). This might be because SIFs and PFs had already achieved a high accuracy and further improvement became difficult. The functions of mRNAs and lncRNAs are significantly different. In general, mRNAs are translated to proteins in a cell, while lncRNAs play important roles in regulation of gene expression. Nucleotide composition, arrangement, and secondary structure are essential for an RNA’s function because they affect its interaction with other molecules. Such functional differences may result in very different sequence and secondary structures of mRNAs and lncRNAs. Indeed, a genome-wide analysis of the stability of lncRNAs and mRNAs shows that mRNAs are more stable than lncRNAs on average, indicating that mRNAs may have more steady secondary structures^40^. As a new type of feature used in machine learning models for lncRNA identification, SASS k-mer score features achieved good performance likely because they recognized secondary structure and sequence variations between mRNAs and lncRNAs.

Five popular algorithms, LR, DT, SVM, RF, and XGBoost were compared in this study. Because XGBoost outperformed other algorithms in performance evaluation, it was used in the LncDC program (Table 1). Besides performance, XGBoost was also faster than SVM during model training. Since the amount of manually curated annotations of RNA transcripts is increasing rapidly, researchers may want to use their own dataset to train models. The advantage of XGBoost in model training speed will enhance the efficiency in customized model training. In addition, LncDC enables parallel processing, which enormously improves the efficiency of model training and lncRNA prediction.

Since the annotated numbers of mRNAs and lncRNAs are usually different in almost all species, balancing of training data is critical for model training. To avoid the loss of important information due to random under-sampling during data balancing, we applied an over-sampling strategy SMOTE, which might also contribute to the excellent performance of LncDC.

We compared the performance of LncDC and six state-of-the-art tools on both H-Test and M-Test. The results showed that LncDC outperformed all the tools on various performance metrics, indicating that the features we extracted and the algorithm we used had powerful abilities in distinguishing mRNAs and lncRNAs. We also showed that LncDC performed better than other tools even if SSFs were excluded. The consistent good performance of the LncDC human model on both H-Test and M-Test suggested that the features we extracted captured the intrinsic variance and discrepancies between mRNAs and lncRNAs.

Integrated with LncDC, we developed a bioinformatics pipeline to identify OS-specific novel lncRNAs from 180 OS samples. Using this pipeline, 97 novel lncRNAs were identified, where 61 of them were in intergenic regions and 36 were in generic areas. Our findings extended the number of OS transcriptome annotations, especially for lncRNAs which may contribute in finding new diagnostic biomarkers or therapeutic targets of OS. Not all of the identified lncRNAs were in intergenic regions, in contrast, several of them could be antisense or pseudogene-derived lncRNAs, suggesting that various types of lncRNAs could be detected by our pipeline. Moreover, by using consensus assemblies from both routes in the pipeline (Supplementary Fig. S5) and validating them by visual check through IGV, the novel lncRNAs we identified are highly reliable. Future works should focus on validating the 97 novel lncRNAs by wet-lab approaches and investigating their biological functions and regulatory mechanisms. Researchers can also use LncDC to identify novel cancer-specific lncRNAs in other cancers.

In this study, we developed a new software named LncDC to classify lncRNAs and mRNAs. We evaluated the performance of LncDC and other six state-of-the-art tools using the datasets formed by the gold standard NCBI RefSeq and GENCODE RNA transcript annotations of humans and mice, respectively. The results showed that LncDC achieved the best performance (~ 98% accuracy) on both H-Test and M-Test, indicating that LncDC has a powerful ability in distinguishing lncRNAs and mRNAs. LncDC is implemented in Python and enables multi-thread running, allowing fast model training and lncRNA prediction. Our pipeline identified 97 novel, high-quality lncRNAs from OS cancer transcriptome data and expanded the annotations of OS transcriptome, providing potential diagnostic biomarkers or targets for OS therapy.

## Methods

### Data set

We collected 57,116 known human reference mRNA transcripts and their CDS annotations from the NCBI RefSeq database (release 99) and 48,479 human lncRNA transcripts from the GENCODE database (human release 34) to build a human dataset with a total of 105,595 transcripts^23,41^. Considering that lncRNAs are longer than 200 nt and the time cost of secondary structure prediction by RNAfold increased a lot when the RNA tends to be very long, we filtered out transcripts with a length of < 200 or > 20,000 nt, besides removing transcripts with invalid nucleotide symbols such as ‘N’ and ‘S’^42^. After such filtrations, there are 105,163 RNA transcripts left in the human dataset for downstream analysis, including 48,165 lncRNAs and 56,998 mRNAs (Supplementary Fig S8A). From the human dataset, we randomly selected 10,000 (~ 21%) lncRNAs and 10,000 (~ 18%) mRNAs respectively to build a human test dataset (H-Test). In order to build a training dataset, we collected the remaining 85,163 RNA transcript from the human dataset and used CD-HIT to remove the transcripts with more than 90% sequence identity against H-Test transcripts^43^. Finally, we obtained 72,237 transcripts, containing 34,539 lncRNAs and 37,698 mRNAs, to build a human train dataset (H-Train) (Supplementary Fig S8B and S8C).

To evaluate the generalization capabilities of LncDC and eliminate the species-specific bias, we also collected 37,866 known mouse reference mRNA transcripts and their corresponding CDS from NCBI RefSeq (release 200) and 18,856 mouse lncRNA transcripts from GENCODE (mouse release M25) to build a mouse dataset^23,41^. After data filtration with the same strategy as in human data, we got 56,443 transcripts in the constructed mouse dataset, which included 18,603 lncRNAs and 37,840 mRNAs (Supplementary Fig S8D). Because the number of annotated lncRNAs is far less than mRNAs in mouse data, to obtain a representative testing dataset for mice, we randomly selected 5000 (~ 27%) lncRNAs and 5000 (~ 13%) mRNAs respectively to build a mouse test dataset (M-Test). Then we removed the transcripts with high sequence identity against the M-Test transcripts as we did in H-Train building, and used the remaining 42,324 transcripts to build a mouse train dataset (M-Train), which includes 12,462 lncRNAs and 29,862 mRNAs (Supplementary Fig S8E and S8F).

To identify novel OS-specific lncRNAs in OS tumor tissues, we downloaded 180 RNA-Seq data of 93 OS patients from the TARGET-OS project. The OS tumor tissues were collected from the primary solid tumors of the patients, followed by paired-end sequencing on the Illumina Genome Analyzer IIx platform (~ 80 million reads per sample on average). The OS RNA-Seq data are available by the accession number ‘phs000468’ in the dbGaP database, and the relevant NCBI BioProject accession number is PRJNA89527. To identify OS-specific lncRNAs, we need to make sure that these lncRNAs can only be detected in OS tumors rather than adjacent normal bone tissues that can be used as control in sampling. Unfortunately, the TARGET-OS project does not have paired RNA-Seq data from normal bone tissues. Thus, we downloaded 9 paired-end RNA-Seq data of 3 adjacent normal bone tissue samples (~ 38 million reads per sample on average; paired-end) as normal controls from the gene expression omnibus (GEO) database (accession number ‘GSE87686’; bioproject number ‘PRJNA345550’)^44^.

### Feature extraction

**Figure 5:**
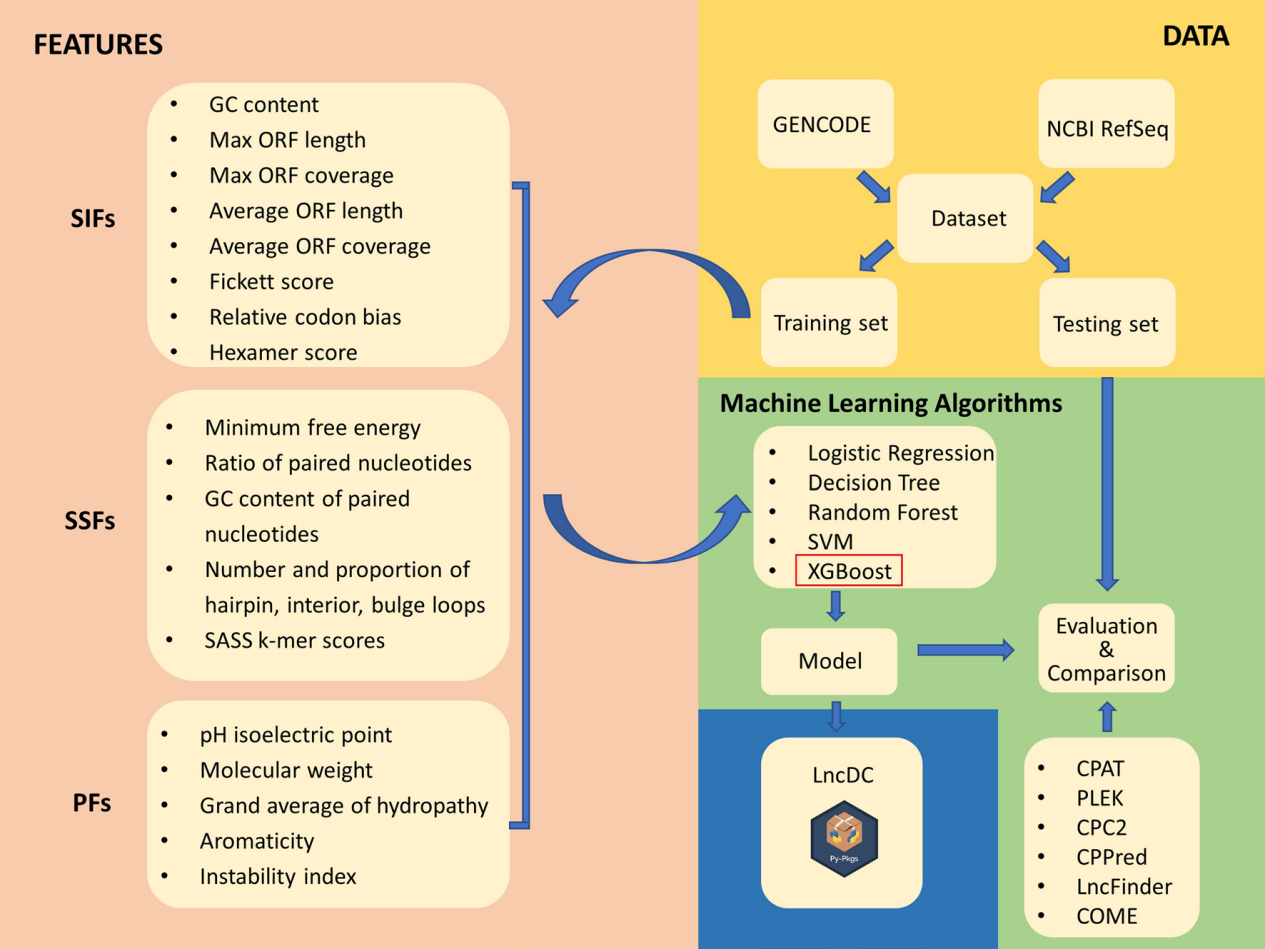
The framework of LncDC design and implementation. The lncRNA and mRNA transcripts data (*yellow*) were downloaded from the GENCODE and NCBI RefSeq databases, respectively. The entire dataset was split into a training dataset and a testing dataset. Features (*pink*), including sequence intrinsic features (SIFs), secondary structure features (SSFs), and protein features (PFs), were extracted from the training dataset. The features and various machine learning algorithms were used to construct different models. The one with the best performance on the testing dataset (i.e., XGBoost) was selected and implemented in a python package named LncDC (*blue*). To illustrate that LncDC has strong power to identify lncRNA transcripts, we benchmarked LncDC against six state-of-the-art tools including CPAT, PLEK, CPC2, CPPred, LncFinder, and COME on the testing dataset. LncDC is flexible for model training and lncRNA prediction.

The framework of LncDC design and implementation is shown in Fig. 5. In LncDC, three feature categories including SIFs, SSFs, and PFs are incorporated to distinguish mRNAs and lncRNAs. The SIFs category contains all the features that are directly derived from RNA primary sequences, such as Fickett score, GC content, max ORF length, etc. In contrast, the SSFs are those extracted from RNA secondary structures and primary sequences of RNA transcripts. At last, the PFs category involves features derived from the putative protein sequence of RNA transcripts.

#### Sequence Intrinsic Features (SIFs)

1. GC content: The frequency of guanine (G) and cytosine (C) in an RNA transcript. Protein coding sequences usually have higher GC content than noncoding sequences, such as 5’ untranslated regions (UTRs) and introns, because they have fewer stop codons in which adenine (A) and thymine (T) are enriched^45^.
  1 $$GC \; content = \frac{N\left( G \right) + N\left( C \right)}{{L_{t} }}$$
  where N(G) and N(C) are the quantity of G and C in an RNA transcript. *L*_t_ denotes the length of an RNA transcript. In general, mRNAs are longer than lncRNAs. A large portion (~ 40%) of the annotated human lncRNAs only have two exons, while most mRNAs have more than two exons^11^.
2. Max ORF length: ORF is a subsequence of an RNA transcript that is likely to be the protein-coding region of the RNA. A long ORF indicates that the RNA transcript has a high possibility to be translated into a protein. mRNAs usually have longer ORFs than lncRNAs. Considering that the CDS and the longest ORF sequence are different in many annotated mRNA transcripts, we adopted features derived from various types of ORF defined in FEELnc to capture the CDS information^27^. As shown in Supplementary Fig S1, ‘Type 0’ (T0) ORF is the same as the conventional defined ORF, which begins with a start codon (e.g., ATG) and ends with a stop codon (e.g., TAG, TAA, or TGA). ‘Type 1’ (T1) ORF only considers start codons, which means it starts with any start codon and stops at the RNA transcript’s end. On the other hand, ‘Type 2’ (T2) ORF only focuses on stop codons that it begins with any non-stop codon and ends with a stop codon. ‘Type 3’ (T3) ORF integrates T1 and T2 from which the longer one is selected. For each type of the defined ORFs, only the longest one in all three reading frames is considered and extracted as the max ORF, such as max ORF T0 (the longest T0 ORF), max ORF T1, max ORF T2, and max ORF T3.
  2 $$\begin{gathered} Max \; ORF \; T0 \; length = L_{orf \; T0} \hfill \\ Max \; ORF \; T1 \; length = L_{orf \; T1} \hfill \\ Max \; ORF \; T2 \; length = L_{orf \; T2} \hfill \\ Max \; ORF \; T3 \; length = L_{orf \; T3} \hfill \\ \end{gathered}$$
3. Max ORF coverage: The length of the max ORF divided by the length of the RNA transcript.
  3 $$\begin{gathered} ORF \; T0 \; coverage = \frac{{L_{orf \; T0} }}{{L_{t} }} \hfill \\ ORF \; T1 \; coverage = \frac{{L_{orf \; T1} }}{{L_{t} }} \hfill \\ ORF \; T2 \; coverage = \frac{{L_{orf \; T2} }}{{L_{t} }} \hfill \\ ORF \; T3 \; coverage = \frac{{L_{orf \; T3} }}{{L_{t} }} \hfill \\ \end{gathered}$$
4. Average ORF length: The average length of all T0 ORFs.
  4 $$Average \; ORF \; length = \frac{1}{n}\mathop \sum \limits_{i = 1}^{n} L_{orf\ T0\ i}$$
5. Average ORF coverage: The average length of all T0 ORFs divided by the length of the RNA transcript.
  5 $$Average \; ORF \; coverage = \frac{Average \; ORF \; length}{{L_{t} }}$$
6. Fickett score: The Fickett score integrates the nucleotide composition and codon usage bias to classify coding and non-coding RNAs^17^. It computes four position values and four content values followed by a weighted summation. The position value is used to represent which codon position a nucleotide prefers. The position values of different nucleotides in an RNA transcript are calculated as follows:
  $$A_{1} = N \left( {base \; A \; in \; position \; 0, 3, 6 \ldots } \right)$$
  $$A_{2} = N \left( {base \; A \; in \; position \; 1, 4, 7 \ldots } \right)$$
  $$A_{3} = N \left( {base \; A \; in \; position \; 2, 5, 8 \ldots } \right)$$
  $$A_{pos} = \frac{{Max \; \left( {A_{1} , A_{2} , A_{3} } \right)}}{{Min \left( {A_{1} , A_{2} , A_{3} } \right) + 1}}$$
  $N()$ denotes the total number of nucleotides in a particular condition. $T_{pos}$, $G_{pos}$, and $C_{pos}$ are calculated in the same way as $A_{pos}$. The content values of different nucleotides in an RNA transcript are calculated as follows:
  $$A_{content} = \frac{{N \left( {base\, A\, in\, an\, RNA\, transcript} \right)}}{{L_{t} }}$$
  $T_{content}$, $G_{content}$ , and $C_{content}$ are computed in the same way. Finally, the four position values and four content values are converted to probabilities of coding ability by using a lookup table. The Fickett score is calculated by multiplying the eight probability values (p) with their corresponding weights (w), where the weights represent the ability that each type of position values or content values classify coding and non-coding sequences successfully^14^.
  6 $$Fickett \; score = \mathop \sum \limits_{i = 1}^{8} p_{i} w_{i}$$
7. Relative codon bias (RCB): The RCB was used to measure the codon triplet usage bias for the ORFs of an RNA transcript^46^. To calculate the RCB value of an ORF, the codon usage bias of codon triplets needs to be figured out and then multiplied together. The codon usage bias ($d_{xyz}$) of a particular codon triplet ($x,y,z$) is calculated as follows:
  $$d_{xyz} = \frac{{f\left( {x, y,z} \right) - f_{1} \left( x \right)f_{2} \left( y \right)f_{3} \left( z \right)}}{{f_{1} \left( x \right)f_{2} \left( y \right)f_{3} \left( z \right)}}$$
  $$f\left( {x,y,z} \right) = \frac{{N\left( {x, y, z} \right)}}{{L_{codon} }}$$
  $$f_{1} \left( x \right) = \frac{{N\left( {base \; x \; in \; position \; 0 \; of \; each \; codon} \right)}}{{L_{codon} }}$$
  where $N\left( {x,y,z} \right)$ is the total number of the codon triplet ($x,y,z$) in the ORF and $L_{codon}$ is the ORF length in codons. $f_{2} \left( y \right)$ and $f_{3} \left( z \right)$ are calculated for the second and third positions of all codon triplets similarly as $f_{1} \left( x \right)$ for the first nucleotide position.
  The RCB value of an RNA transcript ORF is calculated as follows:
  7 $$RCB = \left( {\mathop \prod \limits_{i = 1}^{{L_{codon} }} \left( {1 + d_{xyz}^{i} } \right)} \right)^{1/L} - 1$$
  Here, we calculated RCB values of the four max ORFs including RCB T0, RCB T1, RCB T2, and RCB T3.
8. Hexamer score: A hexamer in an RNA transcript is a subsequence of any six adjacent nucleotides. Since a codon includes three adjacent nucleotides, a hexamer can represent the dependence between two continuous codons in an RNA transcript or two amino acids in a putative protein. We made a hexamer probability lookup table for the LncDC human model by calculating the hexamer probabilities of 34,539 lncRNAs and the CDS of 37,698 mRNAs in H-Train. Because each nucleotide in a hexamer can be A, T, G, or C, there are a total of 4,096 (i.e., 4*4*4*4*4*4 = 4,096) hexamers in the hexamer probability table. The table for the LncDC mouse model was constructed with the M-Train dataset in a similar way to the human lookup table used. For a specific hexamer(X), the coding and non-coding probabilities are calculated as follows:
  $$P\left( X \right)_{coding} = \frac{{N\left( {hexamer \; X \; in \;the \;CDS \;of \;training \;data} \right)}}{{N\left( {total \;hexamers \;in \;the \;CDS \;of \;training \;data} \right)}}$$
  $$P\left( X \right)_{noncoding} = \frac{{N\left( {hexamer\, X\, in\, the\, lncRNAs \,of \,training \,data} \right)}}{{N\left( {total \,hexamers \,in \,the \,lncRNAs \,of \,training \,data} \right)}}$$
  where $N()$ stands for the total number of hexamers in a specific condition. To calculate the value of $N\left( {hexamer \;X \;in \;the \;CDS \;of \;training \;data} \right)$, we used a sliding window with a window size of 6 and a step size of 3 (i.e., one codon size) to scan each CDS of mRNAs in the training data (H-Train or M-Train). However, for $N \left( {hexamer\ X\ in \;the \;lncRNAs \;of \;training \;data} \right)$, we used a moving window with a step size of 1 to scan each lncRNA sequence in the training data because they do not encode any proteins. After creating the hexamer table, the logarithm-likelihood ratio of coding probability over non-coding probability was calculated and then summed up to get the hexamer score of an RNA transcript^14^.
  8 $$Hexamer\; score = \frac{1}{m}\mathop \sum \limits_{i = 1}^{m} log\left( {\frac{{P\left( X \right)_{coding} }}{{P\left( X \right)_{noncoding} }}} \right)$$
  where m stands for the total number of hexamers in an RNA transcript. Because each RNA transcript has three reading frames, we calculated the hexamer score for each frame and selected the highest value among three frames as the final hexamer score for a given RNA transcript.
9. Hexamer score of ORFs: The hexamer score of different types of ORFs. ORF is the potential coding region of an RNA transcript. Both mRNAs and lncRNAs have ORFs, although the ORFs of mRNAs are generally longer than those in lncRNAs. This feature represents the diverse hexamer usage between the ORFs in mRNAs and lncRNAs.

#### Secondary Structure Features (SSFs)

In LncDC, the RNAfold program is used for secondary structure prediction of RNA transcripts^42^. RNAfold uses dynamic programming to predict the secondary structure of an RNA transcript with the minimum free energy (MFE)^47^. The assumption is that when free energy becomes lower, the secondary structure of an RNA transcript becomes more stable. In RNAfold, the secondary structure of an RNA transcript is divided into different types of substructures, including hairpin loop, interior loop, bulge loop, multi loop, and stacking pairs. The free energy values of the substructures are different, and the free energy of an RNA secondary structure is the summation of the free energy of these substructures. Finally, the most stable secondary structure of each RNA transcript is generated by RNAfold and used for downstream feature extraction.

**Figure 6:**
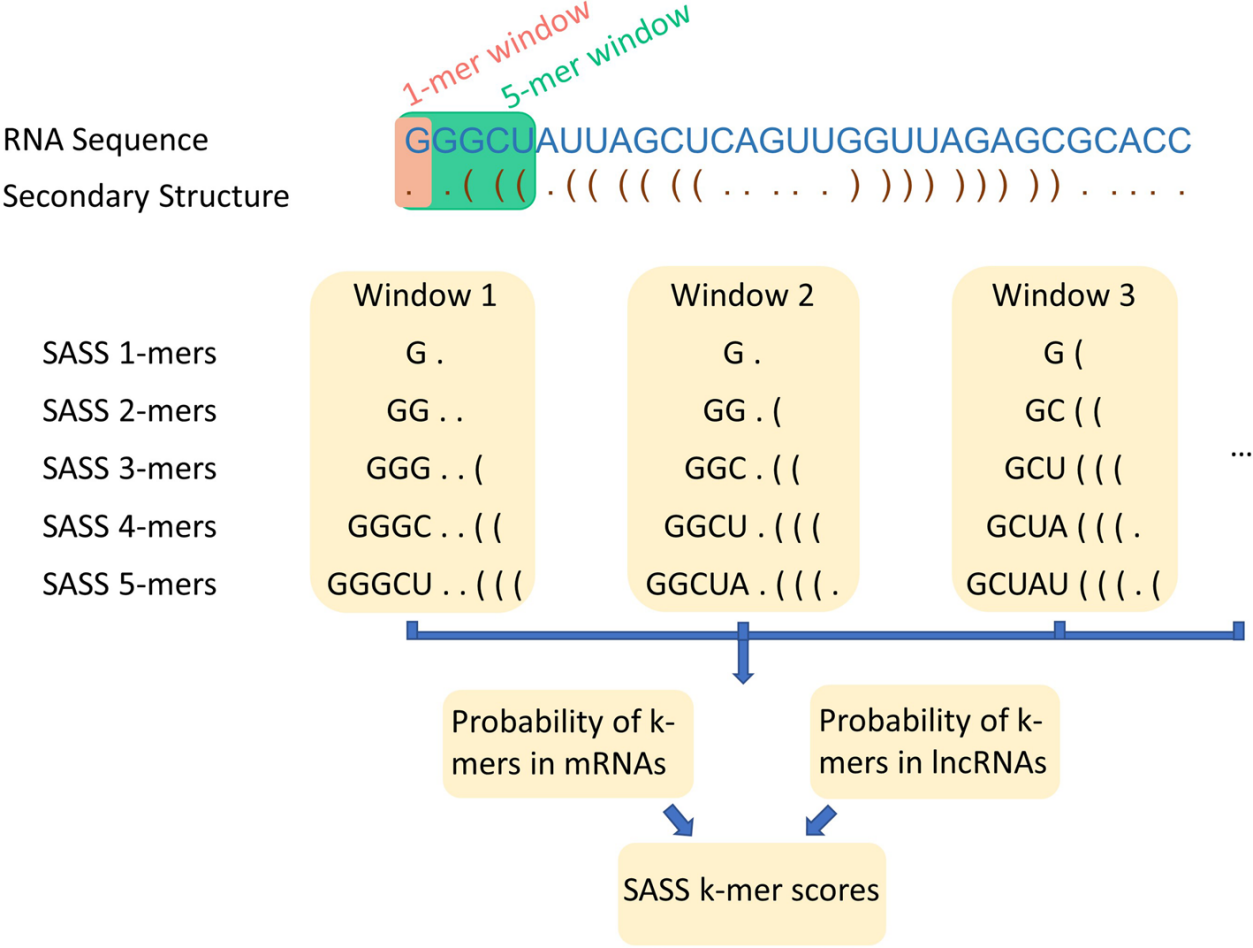
The schema of sequence and secondary structure (SASS) k-mer scores. For any mRNA or lncRNA transcript, we obtained its primary sequence (*in blue font*) and secondary structure (*in brown font*). A sliding window with a length of k and height of 2 was used to scan the sequence and secondary structure of the RNA transcript. The probabilities of the SASS k-mers in mRNAs and lncRNAs were computed, and the SASS k-mer scores were calculated depending on the logarithm-likelihood ratio of the two probabilities.
  We created five SASS k-mer tables to store the counts of the SASS k-mers. Since there are four distinctive nucleotides, ‘A’, ‘U’, ‘G’ and ‘C’ in the primary sequence and three structure forms ‘.’, ‘(‘, and ‘)’ in the secondary structure of the RNA, the total number of combinations will be different among the SASS k-mer tables. The number of distinctive SASS k-mers from SASS 1-mer to SASS 5-mer are 12 (i.e., 4*3 = 12), 144 (i.e., 4*4*3*3 = 144), 1,728 (i.e., 4*4*4*3*3*3 = 1,728), 20,736 (i.e., 4*4*4*4*3*3*3*3 = 20,736) and 248,832 (i.e., 4*4*4*4*4*3*3*3*3*3 = 248,832), respectively. For a specific SASS k-mer(X), the mRNA and lncRNA probabilities are calculated as follows:
  $$P\left( X \right)_{mRNA}^{k} = \frac{{N\left( {SASS \;kmer \;X \;in \;mRNA \;sequences \;of \;training \;data} \right)}}{{N\left( {total \;SASS \;kmers \;in \;mRNA \;sequences \;of \;traning \;data} \right)}}$$
  $$P\left( X \right)_{lncRNA}^{k} = \frac{{N\left( {SASS \;kmer \;X \;in \;lncRNA \;sequences \;training \;data} \right)}}{{N\left( {total \;SASS \;kmers \;in \;lncRNA \;sequences \;of \;traning \;data} \right)}}$$
  where N() stands for the total number of SASS k-mers in a specific condition, and k will be 1, 2, 3, 4, and 5, respectively. Once the SASS k-mer tables were created, the logarithm-likelihood ratio of P(X) of mRNA over P(X) of lncRNA were calculated. We then added them together to get the SASS k-mer score of an RNA transcript.
  12 $$\begin{gathered} {\text{SASS 1-mer score}} = \frac{1}{m}\mathop \sum \limits_{i = 1}^{m} \log \left( {\frac{{P\left( X \right)_{mRNA}^{1} }}{{P\left( X \right)_{lncRNA}^{1} }}} \right) \hfill \\ {\text{SASS 2-mer score}} = \frac{1}{m}\mathop \sum \limits_{i = 1}^{m} \log \left( {\frac{{P\left( X \right)_{mRNA}^{2} }}{{P\left( X \right)_{lncRNA}^{2} }}} \right) \hfill \\ {\text{SASS 3-mer score}} = \frac{1}{m}\mathop \sum \limits_{i = 1}^{m} \log \left( {\frac{{P\left( X \right)_{mRNA}^{3} }}{{P\left( X \right)_{lncRNA}^{3} }}} \right) \hfill \\ {\text{SASS 4-mer score}} = \frac{1}{m}\mathop \sum \limits_{i = 1}^{m} \log \left( {\frac{{P\left( X \right)_{mRNA}^{4} }}{{P\left( X \right)_{lncRNA}^{4} }}} \right) \hfill \\ {\text{SASS 5-mer score}} = \frac{1}{m}\mathop \sum \limits_{i = 1}^{m} \log \left( {\frac{{P\left( X \right)_{mRNA}^{5} }}{{P\left( X \right)_{lncRNA}^{5} }}} \right) \hfill \\ \end{gathered}$$
  where m stands for the total number of k-mers (i.e., 1, 2, 3, 4, or 5) in an RNA transcript.

10. MFE: MFE reveals the stability of a secondary structure. Lower MFE usually represents a more stable secondary structure. Evidence shows that mRNAs are more durable than lncRNAs, suggesting that they tend to have lower MFEs than lncRNAs^40^.
11. Paired ratio: Given the secondary structure of an RNA transcript, we will be able to know which nucleotides have Watson–Crick pairings and which nucleotides are unpaired. If an RNA transcript has a high paired ratio, its secondary structure will be more stable.
  9 $$Paired \;ratio = \frac{{N\left( {Paired \;nucleotieds \;bases} \right)}}{{L_{t} }}$$
12. Number of various loop structures: The number of different types of loops in an RNA transcript, containing hairpin loop (N(H)), interior loop (N(I)), bulge loop (N(B)), and multibranch loop (N(M)). The typical loop structures are shown in Supplementary Fig S9.
13. Coverage of various loop structures: The ratio of the number of different loops divided by the length of an RNA transcript.
  $$C\left( H \right) = \frac{N\left( H \right)}{{L_{t} }}$$
  $$C\left( I \right) = \frac{N\left( I \right)}{{L_{t} }}$$
  $$C\left( B \right) = \frac{N\left( B \right)}{{L_{t} }}$$
  10 $$C\left( M \right) = \frac{N\left( M \right)}{{L_{t} }}$$
14. GC content of paired nucleotides: In the secondary structure of a specific RNA transcript, some of the nucleotides are paired with each other while others are not. This feature measures the GC content of those paired nucleotides. The G-C bond is stronger and more stable than the A-T bond. If an RNA transcript has more G-C pairs, its secondary structure will be more durable.
  11 $$GC \;content \;paired \;nucleotides = \frac{{N\left( {Paired \;G} \right) + N\left( {Paired \;C} \right)}}{{N\left( {paired \;nucleotides} \right)}}$$
15. Sequence and secondary structure (SASS) k-mer scores: We combined a primary sequence and the secondary structure of an RNA transcript to build a two-row array, as shown in Fig. 6. The top row is the primary sequence, and the bottom row is the secondary structure in a dot-bracket format where ‘.’ indicates an unpaired nucleotide, and ‘(‘ and ‘)’ represents paired nucleotides. We used a sliding window with horizontal size 1, vertical size 2, and step size 1 to select SASS 1-mer from the sequence and secondary structure array. As shown in the example in Fig. 6, the first SASS 1-mer in the two-row array is ‘G.’, the second SASS 1-mer is ‘G.’, and the third SASS 1-mer is ‘G(‘, etc. Similar as SASS 1-mer, SASS 2-mers were selected using a sliding window with horizontal size 2, vertical size 2, and step size 1. The first SASS 2-mer is ‘GG..’, the second SASS 2-mer is ‘GG.(‘, etc. The SASS 3-mer, SASS 4-mer, and SASS 5-mer features are extracted in a similar way.

#### Protein features (PFs)

In a cell, mRNAs will be translated to proteins, but lncRNAs are not. However, lncRNAs also have ORFs as mRNAs have, even though their ORFs are usually shorter. We assume that if the longest ORF of a lncRNA transcript can be translated to a protein, this protein will be different from mRNA proteins in many aspects. In LncDC, we used the ProtParam tool from the BioPython package to extract protein features from RNA transcripts^48^.

16. pH isoelectric point (PI): the theoretical pH isoelectric point of a protein. The PI of a protein is the pH value when the protein’s net charge is zero, calculated through the acid dissociation constant (pK) values of amino acids^49^.
17. Molecular weight (MW): the molecular weight of a protein in Daltons.
18. Grand Average of Hydropathy (GRAVY): the hydropathicity of distinctive amino acids is different. The GRAVY value of a specific protein is calculated by summing up the hydropathy values of all the amino acids that the protein contains and being divided by protein length in amino acids^50^.
19. Aromaticity: the amino acids phenylalanine (Phe), tryptophan (Trp), and tyrosine (Tyr) have an aromatic ring structure inside. The aromaticity value of a particular protein is the summation of the numbers of Phe, Trp, and Tyr and then divided by the protein length in amino acids^51^.
20. Instability index (II): The instability index represents the stability of a protein in a test tube. It is calculated by adding up the weighted instability value of each dipeptide where the weights are decided based on the frequency that a dipeptide occurs in experimentally identified unstable and stable proteins^52^.

### Model selection

In this study, various machine learning algorithms were compared, including logistic regression (LR), decision tree (DT), support vector machine (SVM), random forest (RF), and XGBoost. The XGBoost algorithm is implemented by the xgboost package while others are implemented by the scikit-learn package of python^53,54^.

LR fits a logistic curve to a particular dataset, representing the probability of an instance belonging to a specific category, in this case, mRNA or lncRNA^55^. LR is easy to implement and simple for interpretation. However, LR constructs a linear decision boundary so that it is not appropriate for non-linear problems. One solution for this limitation is to increase the number of features such that the dataset can be separated linearly in a high-dimensional feature space.

DT is a tree model that splits the data by several decisions. It has a root node, branches, and leaves. Each path from the root to a leaf defines a rule for the classification of mRNAs and lncRNAs. DT is also easy to interpret and can handle linearly inseparable data. Because DT is a non-parametric model, its prediction does not assume that the data have a specific distribution. Various DT algorithms have been proposed, such as ID3, C4.5, and classification and regression trees (CART). The trees that the CART algorithm builds are binary, suggesting that each node in the tree has two edges^56^. In this project, the CART algorithm is used for DT construction. Although DT has many advantages, it is easy to overfit to the dataset, therefore ensemble models which give the prediction based on several DTs were proposed, such as RF and XGBoost^57^.

As an improvement of DT, a method named bagging constructs many DTs with a bootstrap approach and gives the prediction based on the majority vote of all trees. RF is a special case of bagging that only a subset of features is considered at each tree split^55^. Compared to DT, RF usually has better performance and less overfit to the dataset.

XGBoost is a boosting approach that aggregates several CARTs, where the trees are constructed sequentially. The final prediction value of a specific instance is the summation of the predictions in each tree^53^. To avoid overfitting, XGBoost applies a regularization function to penalize the complexity of the model. Similar to RF, XGBoost considers a subset of features during the tree splitting process as well. XGBoost has been used in biomedical fields for years and achieves good performances in predicting the function or category of biomolecules^58^.

SVM is an approach that uses a hyperplane to separate categories (e.g., mRNAs and lncRNAs) where the minimal distance from the instances to the hyperplane is maximized^55^. It allows non-linear boundaries when using different kernels, such as polynomial kernel and radial kernel, which measure the similarity between two instances. SVM is robust to high-dimensional data, but its training speed is slower than other algorithms when a large number of samples are analyzed^57^.

For each algorithm, several models with distinctive hyperparameters were trained and a tenfold cross-validation approach was used for hyperparameter tuning in H-Train. Different models were evaluated in H-Test, and at last, the XGBoost model was selected and used in LncDC because its overall performance was better than others.

### Feature selection

It was possible that several extracted features were redundant or did not have discriminative classification ability than others. Here, we applied the scikit learn module—recursive feature elimination with cross-validation (RFECV) to select the most powerful features for differentiating mRNAs and lncRNAs^54^. All the 57 extracted features were used to fit a model on H-Train, and the average accuracy score with a tenfold cross-validation was calculated. The features were ranked by their importance scores obtained from the model, followed by eliminating the features with the lowest score. The rest of the features were used to fit a new model and a new average accuracy score was calculated.

### Data preprocessing

In model training, features with a large magnitude may have more impact on the classifiers than the features with a small scale^55^. For instance, a change from 500 to 600 (100 increase) on the ORF length feature is thought to be a more significant shift than the change of a GC content from 0.4 to 0.6 (0.2 gain) by a classifier, while the latter one may have more contribution for differentiating lncRNA and mRNA in reality. To prevent this situation, we standardized the datasets by z-score: z = (x-μ)/σ, where x is the original value in datasets, μ is the mean value of each feature, and σ stands for the standard deviation of each feature. Theoretically, we cannot know the mean value and standard deviation of test data, so we used the mean and standard deviation of the train datasets to standardize the feature values in test datasets.

Data balancing is also critical for machine learning. The model trained from an unbalanced dataset might lead to biased predictions towards the majority class. For instance, if 99% of the RNAs in the dataset are lncRNAs and the classifier simply predicts every RNA is lncRNA, we would see that the classifier can classify 99% of the RNAs correctly, which is heavily misleading. Since the test datasets were already balanced, we would balance the training datasets during the model training process.

The randomly under-sampling strategy for data balancing was used in previously developed lncRNA identification tools, such as FEELnc and CPAT, which randomly dropped a proportion of majority class instances to keep the same instance number as the minority class^14,27^. However, when removing majority class instances, a piece of potentially critical information contributing to classifier learning has also been eliminated.

To avoid the drawback of the randomly under-sampling strategy, we applied the minority over-sampling strategy, SMOTE, to balance training datasets. Instead of oversampling the minority class with replacement, the SMOTE approach synthesizes new minority instances along with the lines that connect a minority instance and its nearest neighbors^29^. The steps of the SMOTE approach are as follows: (1) decide how many new minority instances are required, (2) calculate the *k* nearest neighbors of each minority instance in the feature space, (3) randomly select a minority instance *x*, (4) based on the number of instances required for oversampling, randomly select a certain number of nearest neighbors of *x*, (5) for each *x*-neighbor pair, randomly select a point along the line segment between *x* and its neighbor as the new synthesized minority instance, and (6) repeat the process until the amount of minority class and majority class is balanced.

### Performance evaluation

The performances of LncDC and other tools tested were evaluated by the following standard performance metrics:

$$Sensitivity \left( {Sn} \right) = \frac{TP}{{TP + FN}}$$

$$Specificity \left( {Sp} \right) = \frac{TN}{{TN + FP}}$$

$$Precision \left( {Prec} \right) = \frac{TP}{{TP + FP}}$$

$$Accuracy \left( {Acc} \right) = \frac{TP + TN}{{TP + FP + TN + FN}}$$

$$F - score = 2 * \frac{ Prec * Sn}{{Prec + Sn}}$$

$$MCC = \frac{TP* TN - FP * FN}{{\sqrt {\left( {TP + FP} \right) * \left( {TP + FN} \right) * \left( {TN + FP} \right) * \left( {TN + FN} \right)} }}$$

where TP, true positive, is the number of positive instances (lncRNAs) identified as positive. TN, true negative, is the number of negative instances (mRNAs) identified as negative. FP, false positive, is the number of negative instances identified as positive. FN, false negative, is the number of positive instances identified as negative.

Accuracy evaluates how many instances are correctly classified. Although it is a common metric for evaluating a binary classification model, it cannot comprehensively assess if the model is good or not, especially in an unbalanced test dataset. Sensitivity reflects how many true lncRNAs are picked up by the tool. In contrast, specificity evaluates how many true mRNAs are detected. Precision assesses how many detected lncRNAs are true lncRNAs. F-score and MCC were used to measure the global performance of the tool. F-score is the harmonic mean of precision and sensitivity, and MCC gives a balanced measure even if the number of instances belonging to two classes is different in the test dataset^27^.

In addition, ROC curves were used to evaluate the performances of different classifiers. The ROC curve represents the relationship between true positive rate (TPR) and false positive rate (FPR). Generally, a good classifier has a curve above the TPR = FPR diagonal line and tends to be on the top left corner^59^. If we compare various classifiers, the one with the highest AUC is believed to be the best classifier.

### Transcripts reconstruction of OS and normal tissue RNA-seq samples

180 RNA-seq data collected from 93 tumor samples of OS patients and 9 RNA-Seq data obtained from 3 adjacent normal bone tissue samples were used to conduct transcript reconstruction. Raw data were quality-controlled and cleaned by FastQC v0.11.9 and TrimGalore v0.6.5^60,61^. In our pipeline, we applied two series of widely used approaches (Route 1 and Route 2) for genome mapping and transcript assembling to obtain accurate and consistent RNA transcript assemblies. The pipeline for transcripts reconstruction is shown in Supplementary Fig S5. STAR v2.7.5a was utilized in Route 1 to map the cleaned RNA-Seq reads to human reference genome GRCh38, followed by transcripts assembling through Cufflinks v2.2.1^23,62,63^. We also employed Cuffmerge to merge all of the transcript assemblies to integrated files for the OS and normal control RNA-Seq data, respectively. In Route 2, we used HISAT2 v2.2.0 for reads mapping and StringTie v2.1.2 for assembly reconstruction^64^. To ensure the reconstructed transcript assemblies were expressed in OS or normal tissues, only the assemblies with expression levels in fragments per kilobase million (FPKM) larger than one were selected for downstream analysis.

The expressed RNA transcript assemblies were compared with known human genome annotations by Cuffcompare, such as ENSEMBL and NCBI RefSeq^23,65^. Only the transcript assemblies longer than 200 nt with Cuffcompare generated class codes of ‘U’, ‘I’, ‘O’, and ‘X’ were selected as lncRNA candidates. ‘U’ stands for unknown intergenic transcripts, ‘I’ indicates that the transcripts fall entirely within a reference intron, ‘O’ stands for generic exonic overlap with reference transcripts, and ‘X’ stands for exonic overlap with reference transcripts on the opposite strand (Supplementary Fig S10). The reason we selected transcripts with these codes is that in the GENCODE database most of the lncRNAs are located within the intergenic and protein-coding intronic regions, and the rest of the lncRNAs are overlapped with protein-coding exons on either the same or opposite strand^11^. We also used the CD-HIT program to filter out transcript assemblies that exist in the NONCODE Human database and the transcripts from normal control with 80% identity^43,66^. We conducted filtration with the NONCODE database because it contains lncRNAs obtained from literature mining but may not be verified by GENCODE or NCBI RefSeq yet.

Cufflinks and StringTie were reported to reconstruct many transcript assemblies with a single exon, and most of them are false positives^67^. We removed those transcripts assemblies that only have a single exon to avoid having a large number of false positives in the candidates. Moreover, only the candidate transcript assemblies with the same positions along chromosomes obtained by both Route 1 and Route 2 were kept as high-confidence candidates and fed to LncDC for downstream identification of OS-specific novel lncRNAs.

## Supplementary Information

Supplementary Information.

## Data Availability

The datasets generated and/or analysed during the current study are available in the GenBank repository, [Accession Number: OL779919-OL780015].

## References

[1] Mercer TR, Dinger ME, Mattick JS (2009). Long non-coding RNAs: Insights into functions. Nat. Rev. Genet..

[2] Budak H, Kaya SB, Cagirici HB (2020). Long non-coding RNA in plants in the era of reference sequences. Front. Plant Sci..

[3] Uszczynska-Ratajczak B, Lagarde J, Frankish A, Guigó R, Johnson R (2018). Towards a complete map of the human long non-coding RNA transcriptome. Nat. Rev. Genet..

[4] Cabili MN (2011). Integrative annotation of human large intergenic noncoding RNAs reveals global properties and specific subclasses. Genes Dev..

[5] Statello L, Guo C-J, Chen L-L, Huarte M (2021). Gene regulation by long non-coding RNAs and its biological functions. Nat. Rev. Mol. Cell Biol..

[6] Schmitz SU, Grote P, Herrmann BG (2016). Mechanisms of long noncoding RNA function in development and disease. Cell. Mol. Life Sci..

[7] Wei C-W, Luo T, Zou S-S, Wu A-S (2018). The role of long noncoding RNAs in central nervous system and neurodegenerative diseases. Front. Behav. Neurosci..

[8] Lin C, Yang L (2018). Long noncoding RNA in cancer: Wiring signaling circuitry. Trends Cell Biol..

[9] Bhan A, Soleimani M, Mandal SS (2017). Long noncoding RNA and cancer: A new paradigm. Cancer Res..

[10] Yamkamon V (2020). Urinary PCA3 detection in prostate cancer by magnetic nanoparticles coupled with colorimetric enzyme-linked oligonucleotide assay. EXCLI J..

[11] Derrien T (2012). The GENCODE v7 catalog of human long noncoding RNAs: Analysis of their gene structure, evolution, and expression. Genome Res..

[12] Howald C (2012). Combining RT-PCR-seq and RNA-seq to catalog all genic elements encoded in the human genome. Genome Res..

[13] Kong L (2007). CPC: Assess the protein-coding potential of transcripts using sequence features and support vector machine. Nucleic Acids Res..

[14] Wang L (2013). CPAT: Coding-potential assessment tool using an alignment-free logistic regression model. Nucleic Acids Res..

[15] Zhao J, Song X, Wang K (2016). lncScore: Alignment-free identification of long noncoding RNA from assembled novel transcripts. Sci. Rep..

[16] Crappé J, Van Criekinge W, Menschaert G (2014). Little things make big things happen: A summary of micropeptide encoding genes. EuPA Open Proteom..

[17] Fickett JW (1982). Recognition of protein coding regions in DNA sequences. Nucleic Acids Res..

[18] Fickett JW, Tung CS (1992). Assessment of protein coding measures. Nucleic Acids Res..

[19] Kang Y-J (2017). CPC2: a fast and accurate coding potential calculator based on sequence intrinsic features. Nucleic Acids Res..

[20] Tong X, Liu S (2019). CPPred: Coding potential prediction based on the global description of RNA sequence. Nucleic Acids Res..

[21] Han S (2019). LncFinder: An integrated platform for long non-coding RNA identification utilizing sequence intrinsic composition, structural information and physicochemical property. Brief. Bioinform..

[22] Hu L, Xu Z, Hu B, Lu ZJ (2017). COME: A robust coding potential calculation tool for lncRNA identification and characterization based on multiple features. Nucleic Acids Res..

[23] O’Leary NA (2016). Reference sequence (RefSeq) database at NCBI: Current status, taxonomic expansion, and functional annotation. Nucleic Acids Res..

[24] Chillón I, Marcia M (2020). The molecular structure of long non-coding RNAs: emerging patterns and functional implications. Crit. Rev. Biochem. Mol. Biol..

[25] Yao R-W, Wang Y, Chen L-L (2019). Cellular functions of long noncoding RNAs. Nat. Cell Biol..

[26] Batista GEAPA, Prati RC, Monard MC (2004). A study of the behavior of several methods for balancing machine learning training data. ACM SIGKDD Explor. Newslett..

[27] Wucher V (2017). FEELnc: A tool for long non-coding RNA annotation and its application to the dog transcriptome. Nucleic Acids Res..

[28] Guyon I, Weston J, Barnhill S, Vapnik V (2002). Gene selection for cancer classification using support vector machines. Mach. Learn..

[29] Chawla NV, Bowyer KW, Hall LO, Kegelmeyer WP (2002). SMOTE: Synthetic minority over-sampling technique. J. Artif. Intell. Res..

[30] Mirabello L, Troisi RJ, Savage SA (2009). Osteosarcoma incidence and survival rates from 1973 to 2004: Data from the surveillance, epidemiology, and end results program. Cancer.

[31] Harrison DJ, Geller DS, Gill JD, Lewis VO, Gorlick R (2018). Current and future therapeutic approaches for osteosarcoma. Expert Rev. Anticancer Ther..

[32] Lindsey BA, Markel JE, Kleinerman ES (2016). Osteosarcoma overview. Rheumatol. Ther..

[33] Zhou H (2003). HER-2/ neu expression in osteosarcoma increases risk of lung metastasis and can be associated with gene amplification. J. Pediatr. Hematol. Oncol..

[34] Ebb D (2012). Phase II trial of trastuzumab in combination with cytotoxic chemotherapy for treatment of metastatic osteosarcoma with human epidermal growth factor receptor 2 overexpression: A report from the children’s oncology group. J. Clin. Oncol..

[35] Li Z, Dou P, Liu T, He S (2017). Application of long noncoding RNAs in osteosarcoma: Biomarkers and therapeutic targets. Cell. Physiol. Biochem..

[36] Sun J (2016). Long noncoding RNA FGFR3-AS1 promotes osteosarcoma growth through regulating its natural antisense transcript FGFR3. Mol. Biol. Rep..

[37] Wang Y (2015). A novel long non-coding RNA, hypoxia-inducible factor-2α promoter upstream transcript, functions as an inhibitor of osteosarcoma stem cells in vitro. Mol. Med. Rep..

[38] Li X (2021). Overexpression of GUCY1A2 correlates with poor prognosis in gastric cancer patients. Front. Oncol..

[39] Kung JTY, Colognori D, Lee JT (2013). Long noncoding RNAs: Past, present, and future. Genetics.

[40] Clark MB (2012). Genome-wide analysis of long noncoding RNA stability. Genome Res..

[41] Frankish A (2019). GENCODE reference annotation for the human and mouse genomes. Nucleic Acids Res..

[42] Lorenz R (2011). ViennaRNA Package 2.0. Algorithms Mol. Biol..

[43] Li W, Godzik A (2006). Cd-hit: A fast program for clustering and comparing large sets of protein or nucleotide sequences. Bioinforma. Oxf. Engl..

[44] Scott MC (2018). Comparative transcriptome analysis quantifies immune cell transcript levels, metastatic progression and survival in osteosarcoma. Cancer Res..

[45] Wuitschick JD, Karrer KM (1999). Analysis of genomic G + C content, codon usage, initiator codon context and translation termination sites in tetrahymena thermophila. J. Eukaryot. Microbiol..

[46] Roymondal U, Das S, Sahoo S (2009). Predicting gene expression level from relative codon usage bias: An application to *Escherichia coli* genome. DNA Res Int. J. Rapid Publ. Rep. Genes Genomes.

[47] Zuker M, Stiegler P (1981). Optimal computer folding of large RNA sequences using thermodynamics and auxiliary information. Nucleic Acids Res..

[48] Cock PJA (2009). Biopython: Freely available Python tools for computational molecular biology and bioinformatics. Bioinformatics.

[49] Bjellqvist B (1993). The focusing positions of polypeptides in immobilized pH gradients can be predicted from their amino acid sequences. Electrophoresis.

[50] Kyte J, Doolittle RF (1982). A simple method for displaying the hydropathic character of a protein. J. Mol. Biol..

[51] Lobry JR, Gautier C (1994). Hydrophobicity, expressivity and aromaticity are the major trends of amino-acid usage in 999 Escherichia coli chromosome-encoded genes. Nucleic Acids Res..

[52] Guruprasad K, Reddy BVB, Pandit MW (1990). Correlation between stability of a protein and its dipeptide composition: A novel approach for predicting in vivo stability of a protein from its primary sequence. Protein Eng. Des. Sel..

[53] Chen, T. & Guestrin, C. XGBoost: A Scalable Tree Boosting System. In *Proceedings of the 22nd ACM SIGKDD International Conference on Knowledge Discovery and Data Mining* 785–794 (Association for Computing Machinery, 2016). doi:10.1145/2939672.2939785.

[54] Pedregosa F (2011). Scikit-learn: Machine learning in Python. J. Mach. Learn. Res..

[55] James G, Witten D, Hastie T, Tibshirani R (2013). An Introduction to Statistical Learning.

[56] Rokach L, Maimon O (2005). Top-down induction of decision trees classifiers—a survey. IEEE Trans. Syst. Man Cybern. Part C Appl. Rev..

[57] Singh, A., Thakur, N. & Sharma, A. A review of supervised machine learning algorithms. In *2016 3rd International Conference on Computing for Sustainable Global Development (INDIACom)* 1310–1315 (2016).

[58] Babajide Mustapha I, Saeed F (2016). bioactive molecule prediction using extreme gradient boosting. Molecules.

[59] Powers, D. M. W. Evaluation: From precision, recall and F-measure to ROC, informedness, markedness and correlation. (2020).

[60] Andrews, S. FastQC: A quality control tool for high throughput sequence data [Online]. *Available Online Httpwwwbioinformaticsbabrahamacukprojectsfastqc* (2010) https://qubeshub.org/resources/fastqc.

[61] Martin M (2011). Cutadapt removes adapter sequences from high-throughput sequencing reads. EMBnet. J..

[62] Dobin A (2013). STAR: Ultrafast universal RNA-seq aligner. Bioinformatics.

[63] Trapnell C (2012). Differential gene and transcript expression analysis of RNA-seq experiments with TopHat and Cufflinks. Nat. Protoc..

[64] Pertea M, Kim D, Pertea GM, Leek JT, Salzberg SL (2016). Transcript-level expression analysis of RNA-seq experiments with HISAT, StringTie and Ballgown. Nat. Protoc..

[65] Yates AD (2020). Ensembl 2020. Nucleic Acids Res..

[66] Zhao Y (2016). NONCODE 2016: An informative and valuable data source of long non-coding RNAs. Nucleic Acids Res..

[67] Sahraeian SME (2017). Gaining comprehensive biological insight into the transcriptome by performing a broad-spectrum RNA-seq analysis. Nat. Commun..

