# Supplementary material for: LncDC: a machine learning-based tool for long non-coding RNA detection from RNA-Seq data
Source: Sci Rep. 2022 Nov 9;12:19083. doi: 10.1038/s41598-022-22082-7 (PMC9646749; doi:10.1038/s41598-022-22082-7)
Supplement: Supplementary file 1 — Supplementary Information. [file 41598_2022_22082_MOESM1_ESM.docx]

**LncDC: a machine learning-based tool for long non-coding RNA detection from RNA-Seq data**

Minghua Li^*^ and Chun Liang^*^

Department of Biology, Miami University, Oxford, OH, 45056, USA

**Supplementary Materials**

[Supplementary Figure S1. Schema of four types of ORFs defined in LncDC 2](#_Toc112423522)

[Supplementary Figure S2. The distribution of the hexamer score 3](#_Toc112423523)

[Supplementary Figure S3. The distribution of the relative codon bias (RCB) score 4](#_Toc112423524)

[Supplementary Figure S4. The ROC curves and AUC values for distinctive models on H-Test 5](#_Toc112423525)

[Supplementary Figure S5. An overview of our bioinormatics pipeline for RNA transcripts reconstruction and identification of OS-specific novel lncRNAs 6](#_Toc112423526)

[Supplementary Figure S6. Characteristics of the newly identified OS-specific lncRNAs 7](#_Toc112423527)

[Supplementary Figure S7. The screenshots of the newly identified OS-specific lncRNA gene 8](#_Toc112423528)

[Supplementary Figure S8. Composition of the RNA transcripts in different datasets 9](#_Toc112423529)

[Supplementary Figure S9. Illustration of RNA secondary Loop structures 10](#_Toc112423530)

[Supplementary Figure S10. Illustration of RNA transcript location class codes 11](#_Toc112423531)

[Supplementary Table S1. Performance of different feature subsets on H-Test 12](#_Toc112423532)

[Supplementary Table S2. The selected 28 features 12](#_Toc112423533)


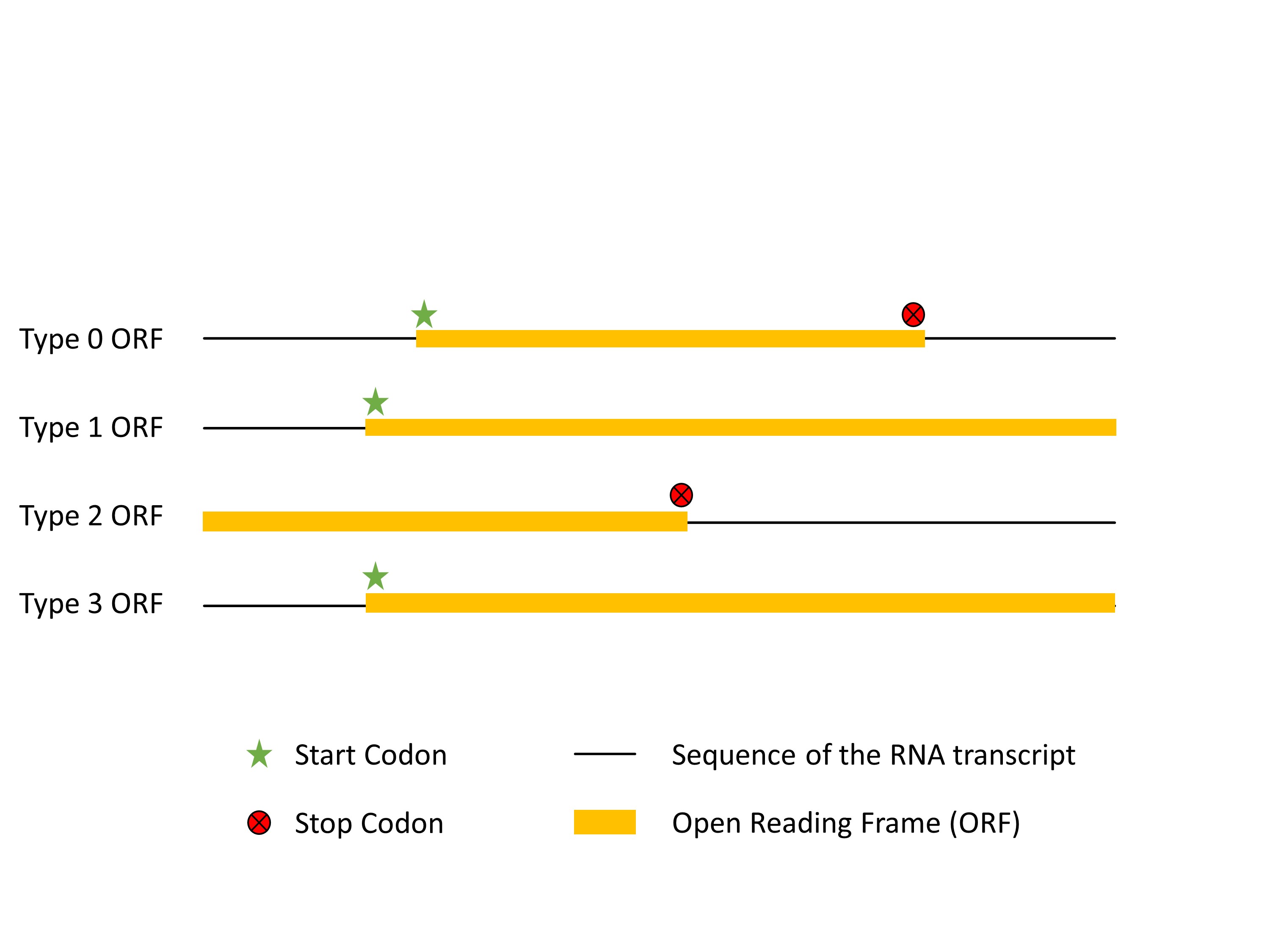


## Supplementary Figure S1. Schema of four types of ORFs defined in LncDC, adopted from FEELnc^1^. Type 0 ORF begins with a start codon and ends with a stop codon. Type 1 ORF starts with a start codon and ends at the end of the RNA transcript. Type 2 ORF starts at the beginning of the RNA transcript and ends with a stop codon. Type 3 ORF is an integration of type 1 and type 2 ORFs from which the longer one is selected.


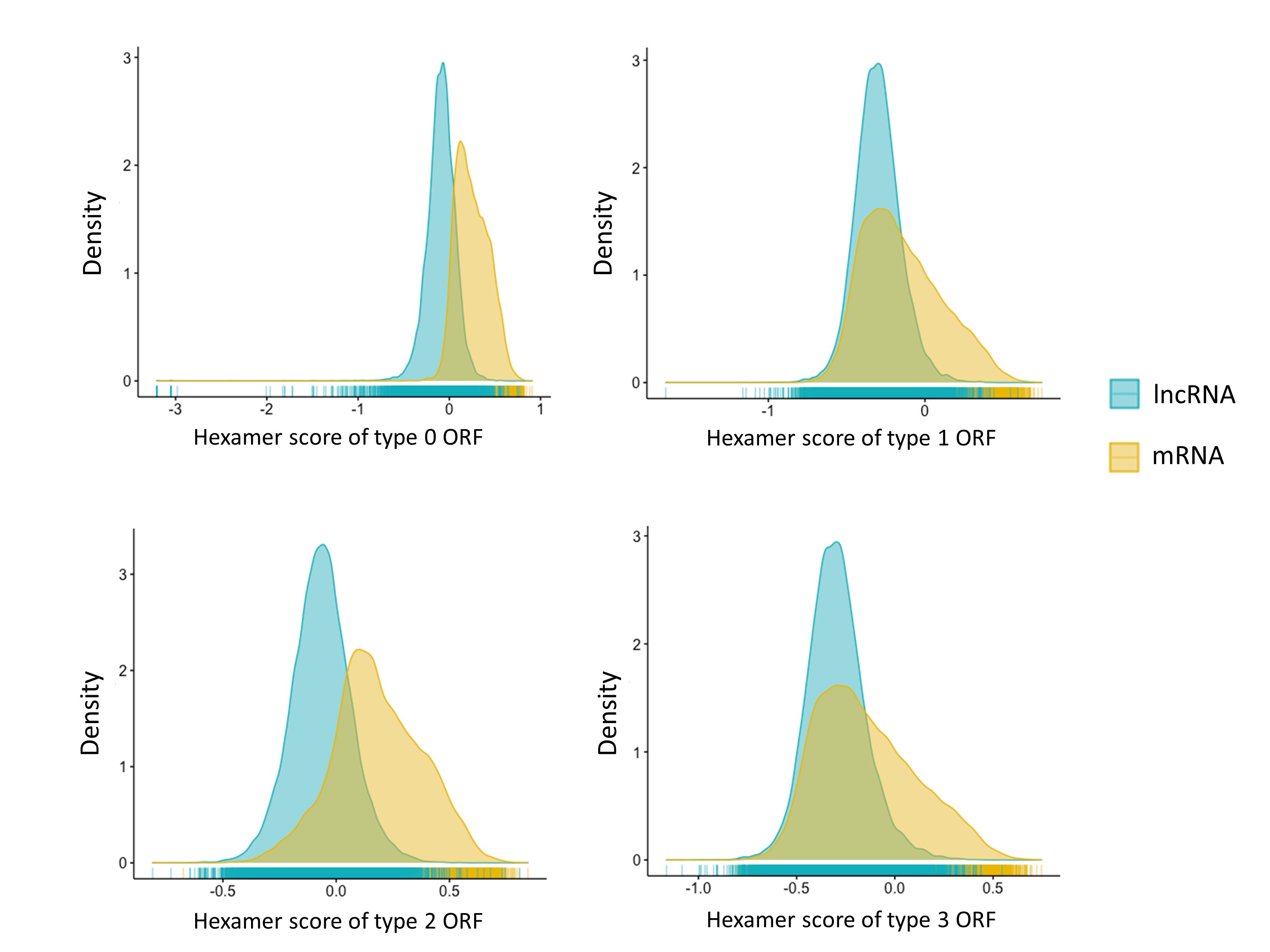


## Supplementary Figure S2. The distribution of the hexamer score of four types (type 0, 1, 2, and 3) of ORFs for lncRNAs and mRNAs in the H-Train dataset. The x-axis is the hexamer score of ORFs and the y-axis is density.


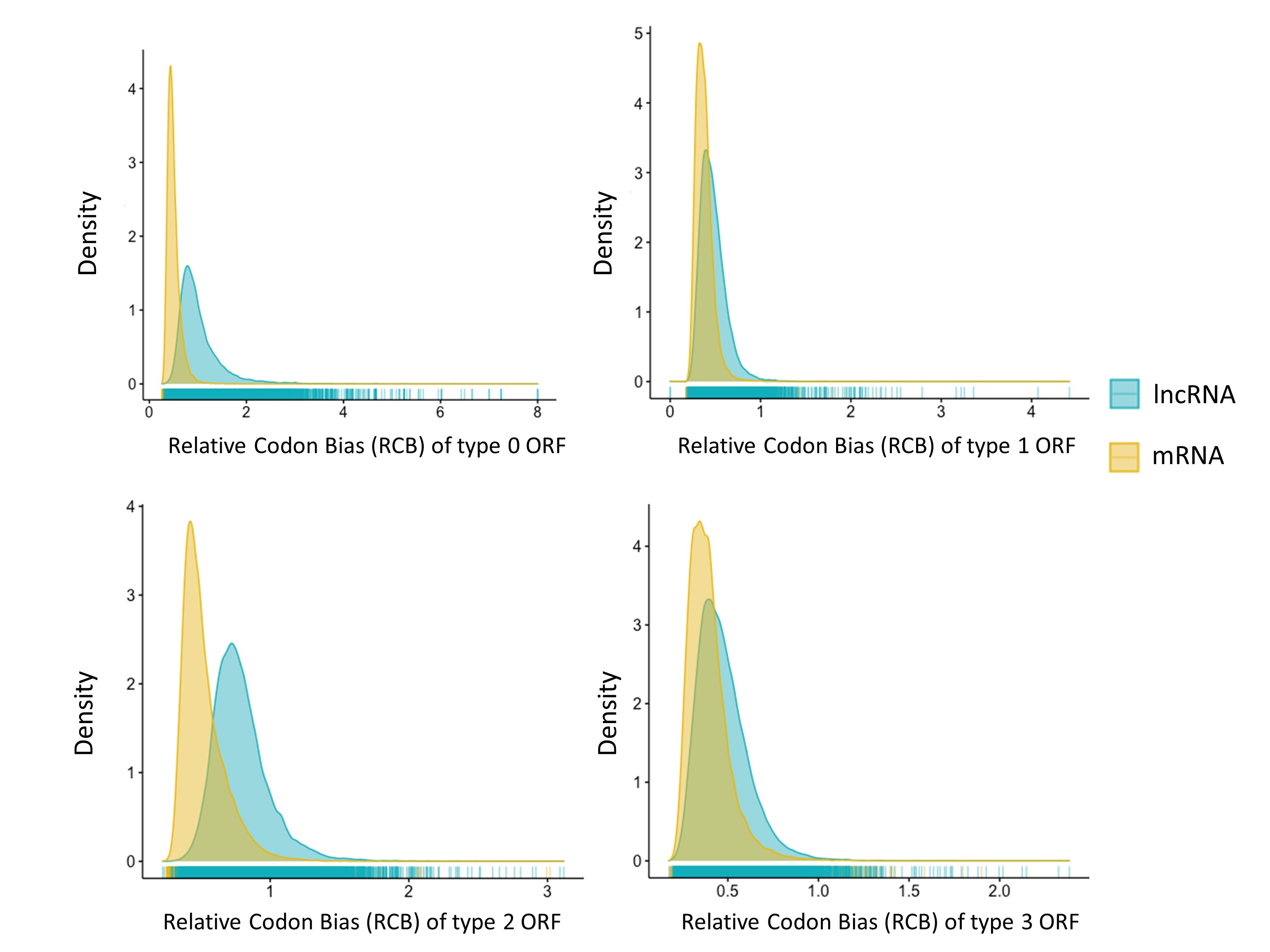


## Supplementary Figure S3. The distribution of the relative codon bias (RCB) score of four types (type 0, 1, 2, and 3) of ORFs for lncRNAs and mRNAs in H-Train. The x-axis is the RCB score of ORFs and the y-axis is density.


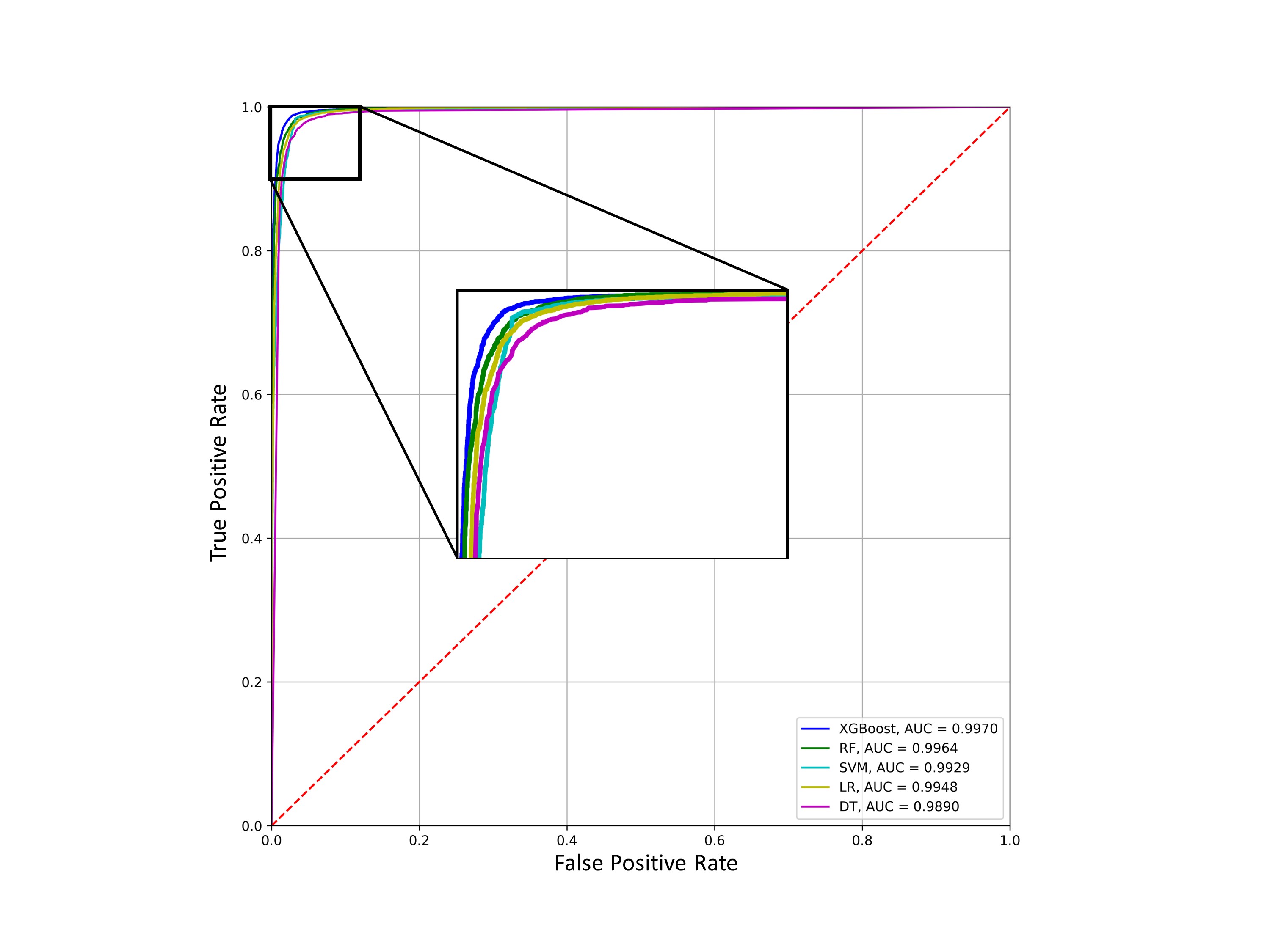


## Supplementary Figure S4. The ROC curves and AUC values for distinctive models on H-Test. The XGBoost model has the highest AUC value while the DT model has the lowest.


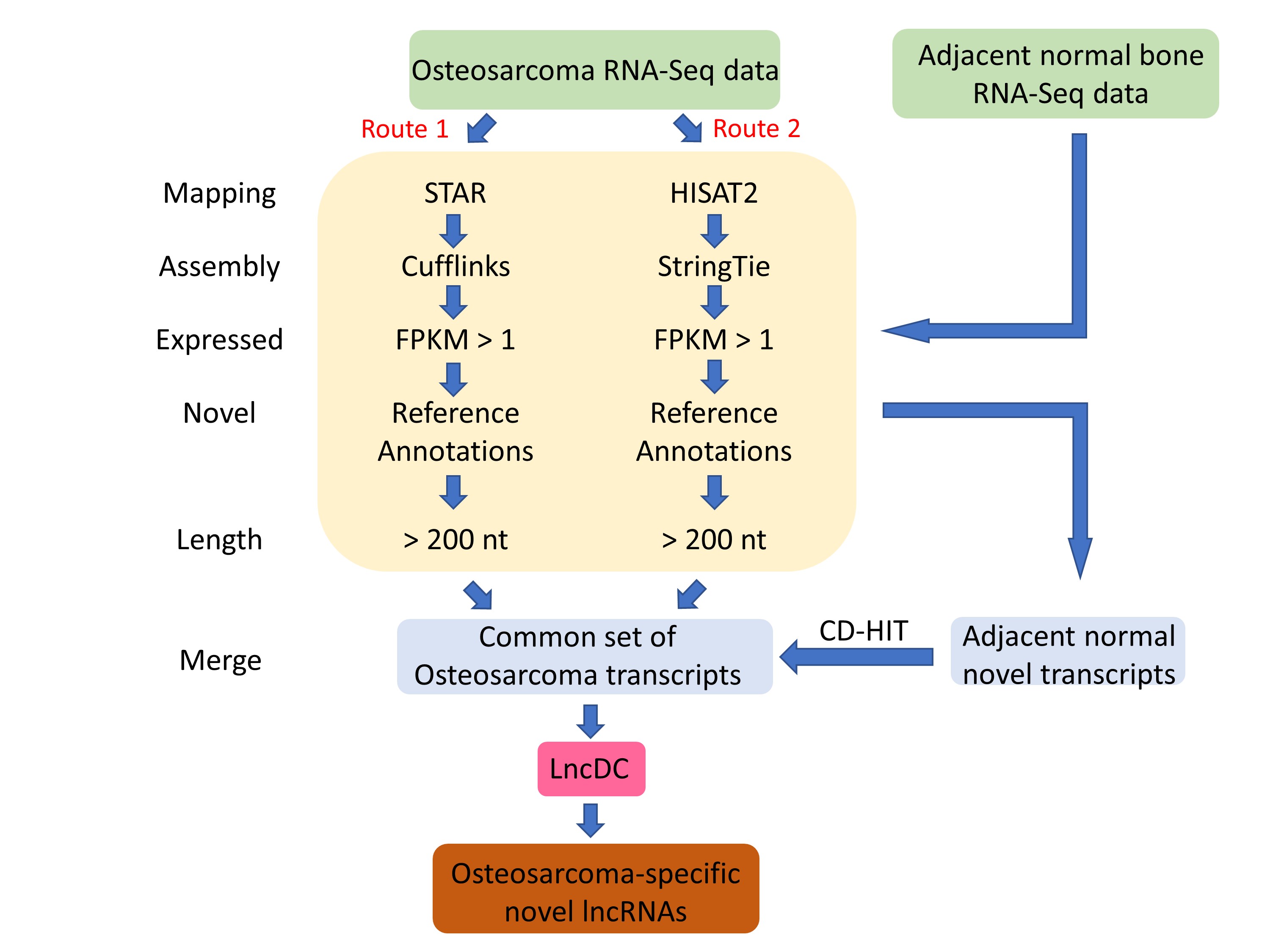


## Supplementary Figure S5. An overview of our bioinormatics pipeline for RNA transcripts reconstruction and identification of OS-specific novel lncRNAs. Input files including both OS and normal control RNA-Seq data were fed to the pipeline, respectively. The cleaned reads were mapped to the human reference genome by either STAR from Route 1 or HISAT2 from Route 2, followed by transcripts assembled by Cufflinks or StringTie, respectively. The assembled RNA transcripts with FPKM less than 1 and lengths less than 200 nt were filtered out. In addition, the transcripts that were annotated in the GENCODE, NCBI RefSeq, and NONCODE databases were removed. After the filtration steps, the OS transcripts with the same genomic positions predicted by both Route 1 and Route 2 approaches were extracted as the combined set of OS transcripts. The OS transcripts in the adjacent normal bone tissues of the same patients were removed from the combined set by CD-HIT with a particular identity threshold. The combined set of OS transcripts were fed to the LncDC program, which would generate an output file that contains OS-specific novel lncRNAs.


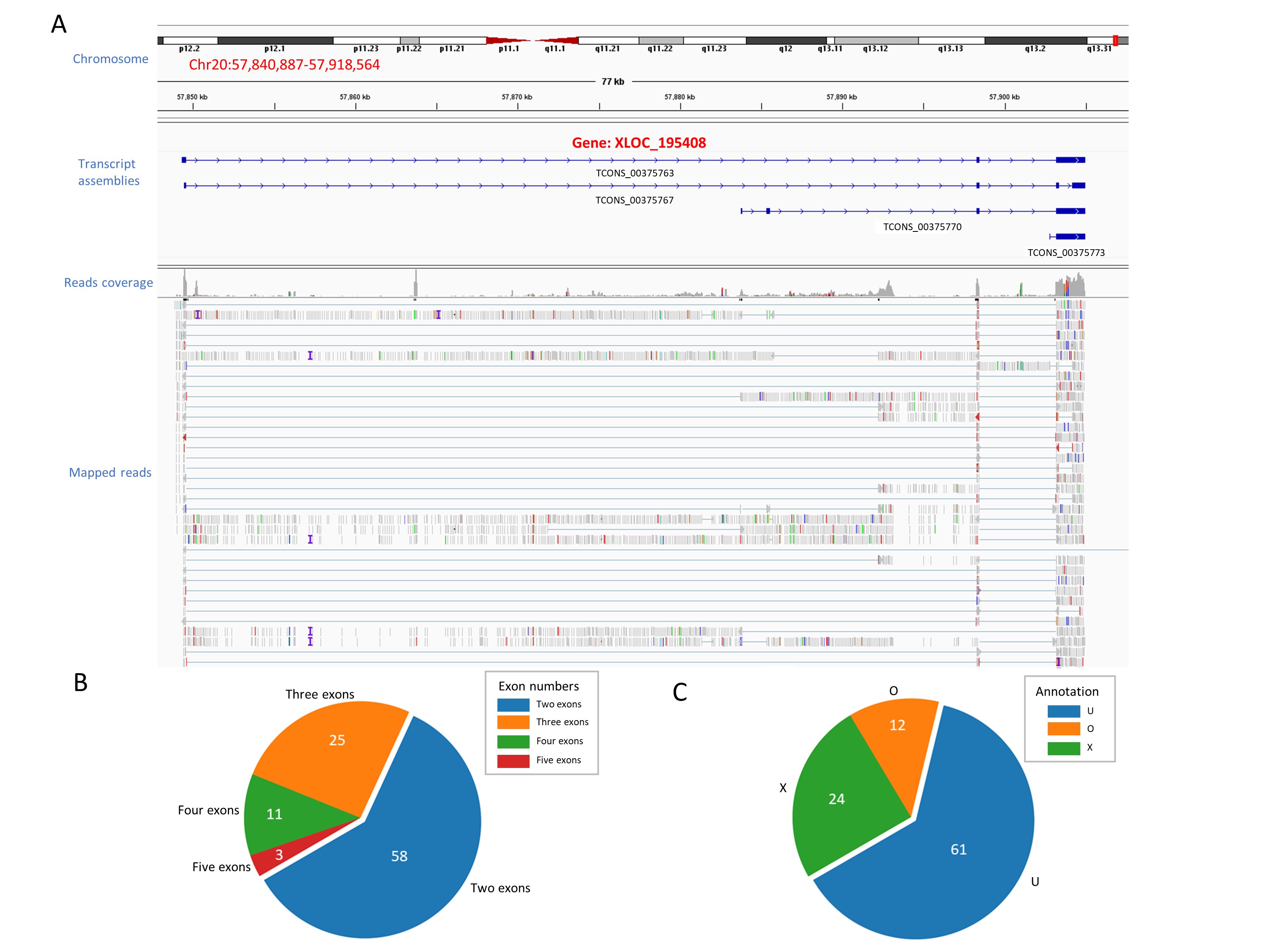


## Supplementary Figure S6. Characteristics of the newly identified OS-specific lncRNAs. (**A**) The screenshot of the newly identified OS-specific lncRNA gene XLOC_195408 and its four RNA transcripts in the integrative genomics viewer (IGV). The gene is located in the intergenic region of human chromosome 20. The solid blue blocks represent the exon regions, and the solid lines are the intron regions. The mapped sequencing reads provided evidence for the RNA transcript assemblies, and the reads coverage peaks indicate valid exons. (**B**) Composition of the exon numbers of the lncRNAs. Most of the novel OS-specific lncRNAs have two exons, while the others have three or more exons. (**C**) Composition of the annotated locations of the lncRNAs. Most of the novel OS-specific lncRNAs are within the intergenic regions (U) of the genome. In contrast, others are located in the intragenic areas that overlap with the reference exons on the same strand (O) or opposite strand (X).


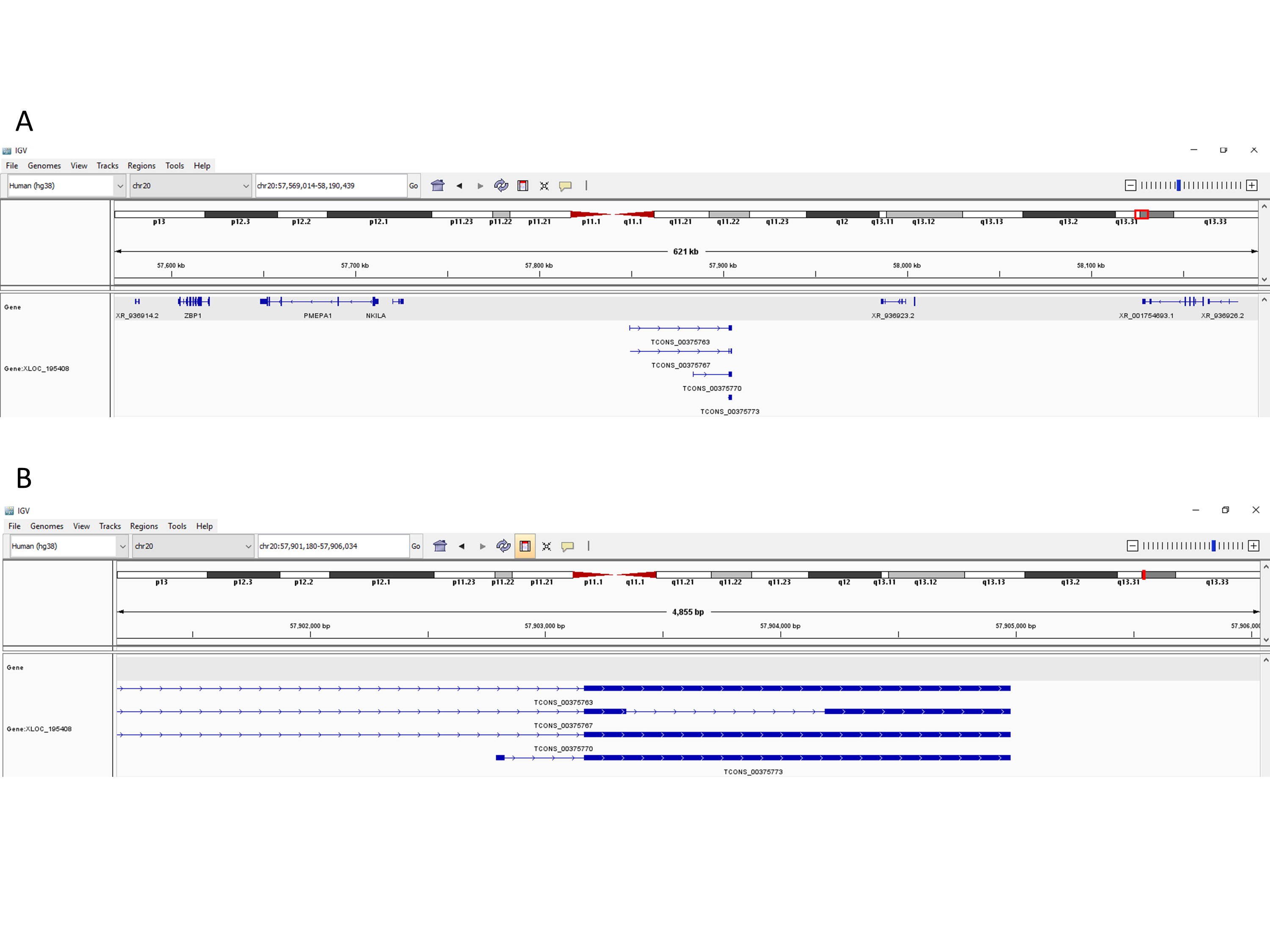


## Supplementary Figure S7. The screenshots of the newly identified OS-specific lncRNA gene XLOC_195408 and its four RNA transcript isoforms in the integrative genomics viewer (IGV). (A) The screenshot shows that the XLOC_195408 gene is not overlapped with other existing genes, indicating that it is located in the intergenic region. (B) The magnified screenshot shows the exons of four transcript isoforms at the 3’ end. Transcript TCONS_00375763 has three exons. TCONS_00375767 has four exons and the two exons at the 3’ end overlapped with TCONS_00375763 with an intron inside. TCONS_00375770 has four exons and its last exon is the same as the one of TCONS_00375763. TCONS_00375773 is the shortest and it only has two exons. Its first exon is relatively small in size than its second exon, and the second exon is the same as the ones of TCONS_00375763 and TCONS_00375770.


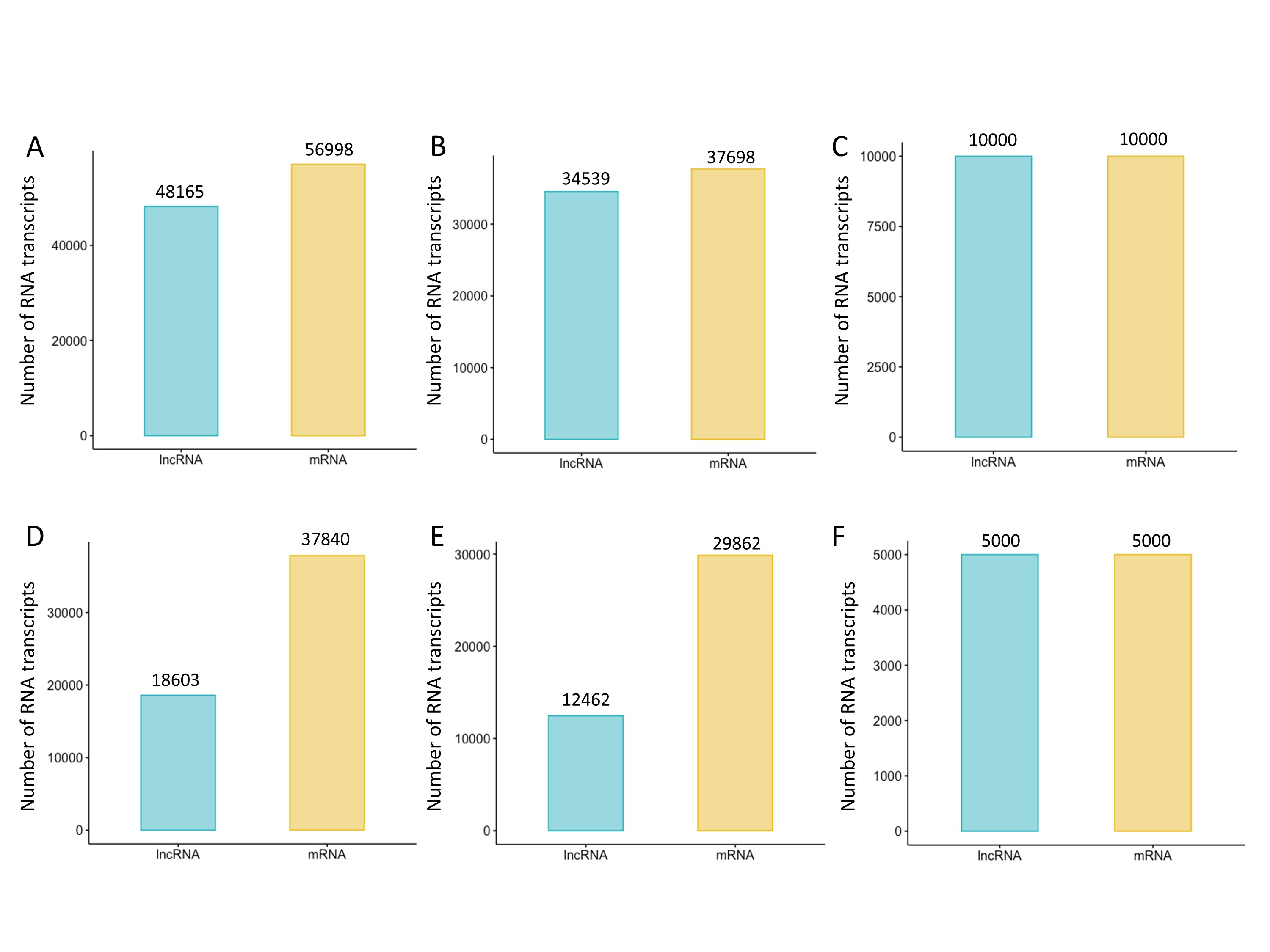


## Supplementary Figure S8. Composition of the RNA transcripts in different datasets. (A) Human dataset. (B) Human training dataset (H-Train). (C) Human testing dataset (H-Test). (D) Mouse dataset. (E) Mouse training dataset (M-Train). (F) Mouse testing dataset (M-Test).


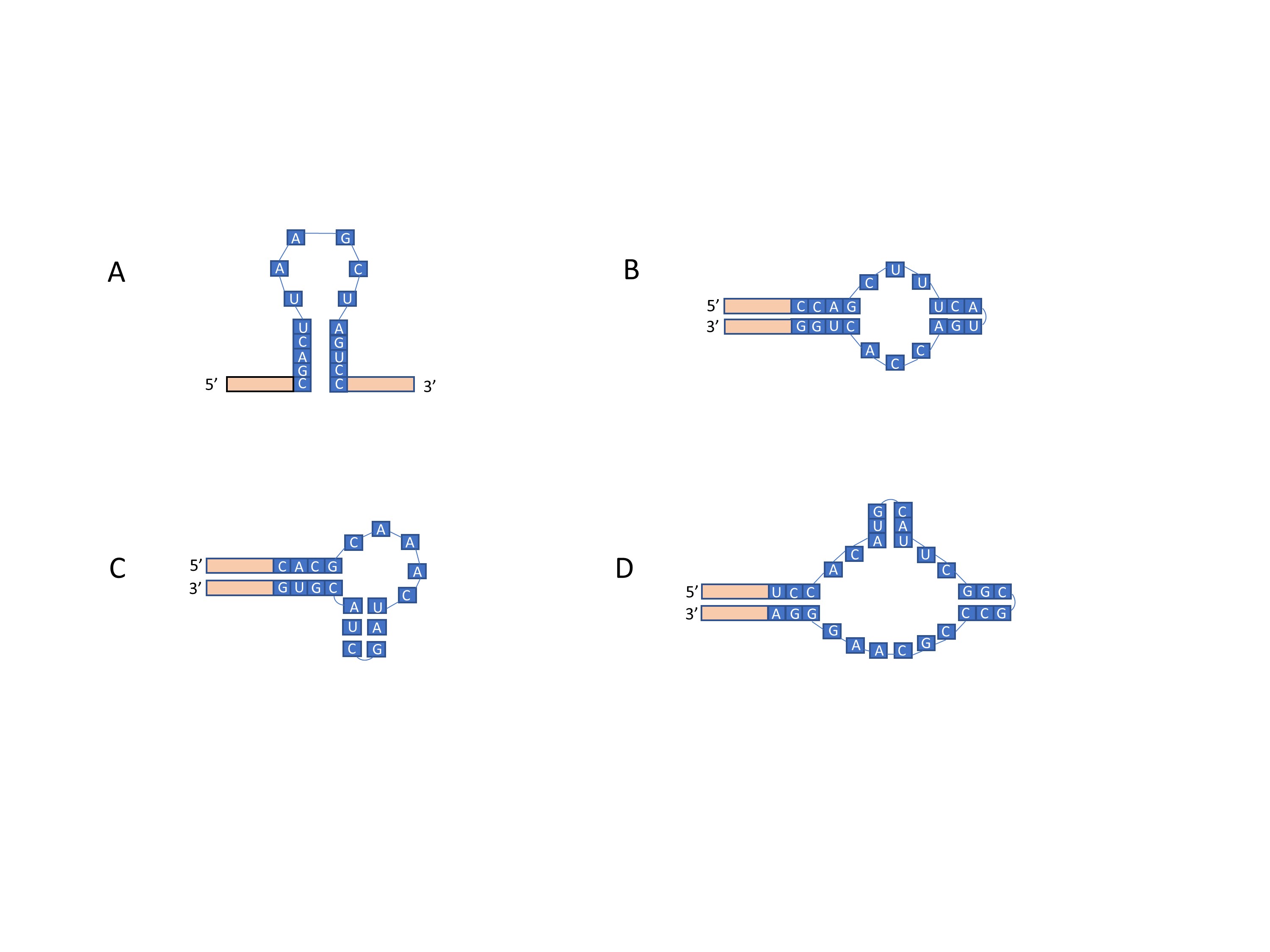


## Supplementary Figure S9. Illustration of RNA secondary Loop structures. (A) Hairpin loop. (B) Interior loop. (C) Bulge loop. (D) Multibranch loop.


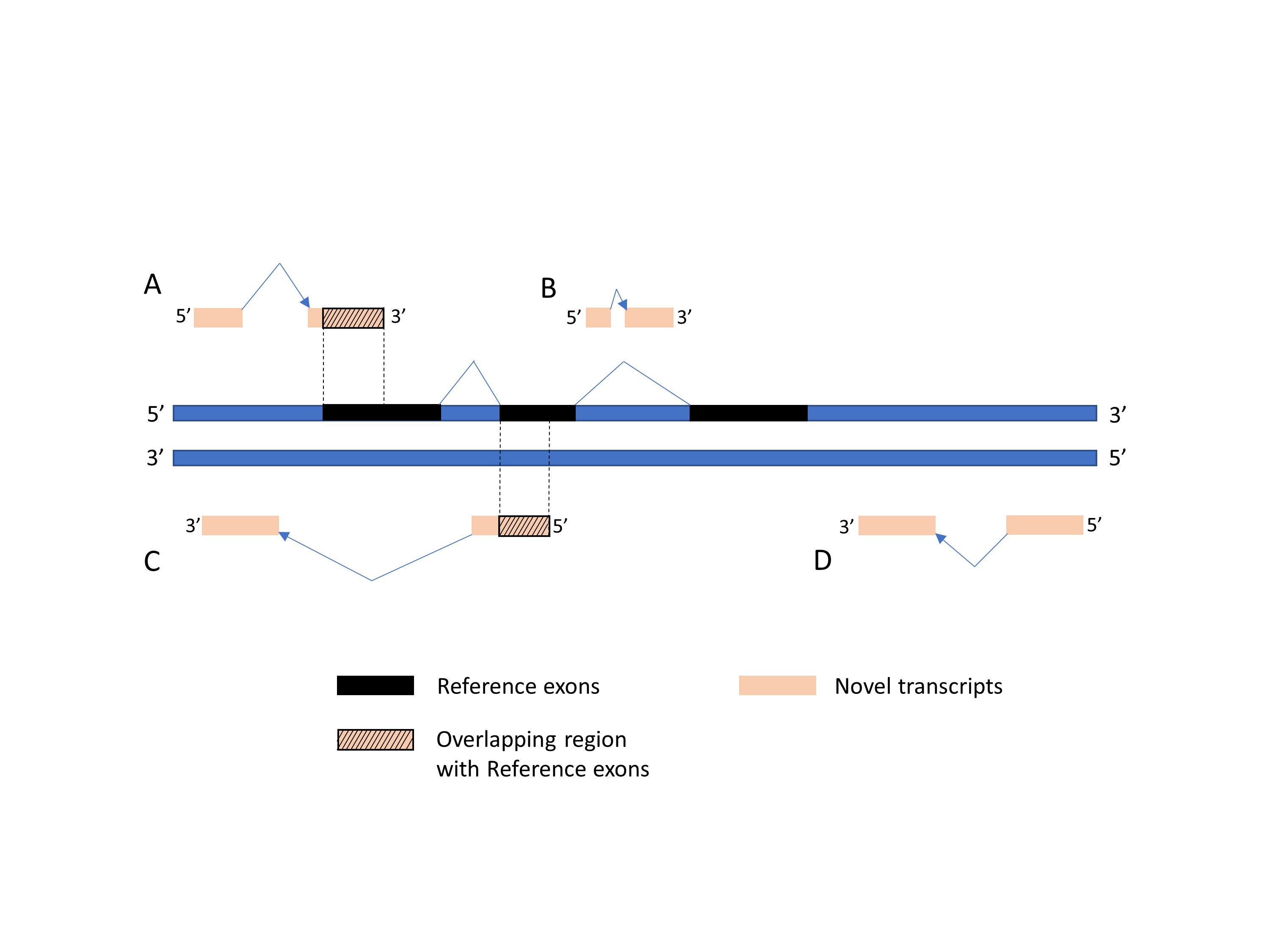


## Supplementary Figure S10. Illustration of RNA transcript location class codes. (A) class code ‘O’: the novel transcript overlaps with reference exons on the same strand. (B) class code ‘I’: the novel transcript is completely within a reference intron. (C) class code ‘X’: the novel transcript overlaps with reference exons on the opposite strand. (D) class code ‘U’: the novel transcript does not overlap with any reference genes.

## Supplementary Table S1. Performance of different feature subsets on H-Test. Bold values correspond to the highest values of each metric.

| Feature subset | $Sensitivity$ | $Specificity$ | $Precision$ | $Accuracy$ | $F-score$ | $MCC$ |
| --- | --- | --- | --- | --- | --- | --- |
| Top 53 features | **0.9860** | 0.9744 | 0.9746 | **0.9802** | **0.9803** | **0.9604** |
| Top 28 features | 0.9812 | **0.9786** | **0.9786** | 0.9799 | 0.9799 | 0.9598 |
| Top 22 features (SSFs excluded) | 0.9822 | 0.9745 | 0.9746 | 0.9783 | 0.9784 | 0.9567 |
| The 6 selected SSFs | 0.8362 | 0.8998 | 0.8929 | 0.8680 | 0.8636 | 0.7374 |

## Supplementary Table S2. The selected 28 features come from three feature categories: SIFs, SSFs, and PFs. Features in each category are listed in descending order of their importance ranks.

| **Sequence intrinsic features (SIFs)** | **Secondary structure features (SSFs)** | **Protein features (PFs)** |
| --- | --- | --- |
| Max ORF T0 length | GC content of paired nucleotides | ORF T0 MW |
| Hexamer score ORF T0 | SASS 5-mer score | ORF T1 MW |
| ORF T3 coverage | SASS 2-mer score | ORF T0 PI |
| GC content | SASS 3-mer score | ORF T3 MW |
| Hexamer score ORF T2 | SASS 1-mer score | ORF T0 aromaticity |
| Fickett score | SASS 4-mer score | ORF T0 instability |
| Hexamer score ORF T1 |  | ORF T1 instability |
| Max ORF T1 length |  | ORF T2 MW |
| RCB T1 |  |  |
| Hexamer score ORF T3 |  |  |
| RCB T0 |  |  |
| ORF T1 coverage |  |  |
| Max ORF T2 length |  |  |
| ORF T0 coverage |  |  |

**References:**

1. Wucher V, Legea1. Wucher, V. *et al.* FEELnc: a tool for long non-coding RNA annotation and its application to the dog transcriptome. *Nucleic Acids Res.* **45**, e57 (2017).
